# Differentiation of Indigenous *Saccharomyces cerevisiae* Wine Strains by Oenological and Proteomic Characterization

**DOI:** 10.3390/ijms27219419

**Published:** 2026-10-22

**Authors:** Daniela Braconi, Simona Guerrini, Francesca Girolamo, Simone Costagli, Alessandro Paffetti, Alessandro Armini, Sara Draghi, Gabriella Jacomelli, Andrea Bernini, Daniela Grasso, Viola Galli, Donatella Ganucci, Lisa Granchi, Annalisa Santucci

**Affiliations:** 1 Department of Biotechnology, Chemistry and Pharmacy (DBCF), University of Siena, Via Aldo Moro 2, 53100 Siena, Italy; francesca.girolamo@student.unisi.it (F.G.); simone.costagli@unisi.it (S.C.); alessandro.paffetti@unisi.it (A.P.); alessandro.armini@unisi.it (A.A.); sara.draghi@unisi.it (S.D.); gabriella.iacomelli@unisi.it (G.J.); andrea.bernini@unisi.it (A.B.); daniela.grasso2@unisi.it (D.G.); annalisa.santucci@unisi.it (A.S.); 2 Department of Agriculture, Food, Environment and Forestry (DAGRI), University of Florence, Via San Bonaventura, 13-50145 Florence, Italy; simona.guerrini@unifi.it (S.G.); donatella.ganucci@unifi.it (D.G.); lisa.granchi@unifi.it (L.G.); 3 Department of Molecular Medicine and Development, University of Siena, 53100 Siena, Italy; 4 Laboratory of Molecular Microbiology and Biotechnology, Department of Medical Biotechnologies, University of Siena, 53100 Siena, Italy; 5 FoodMicroTeam s.r.l., ex Academic Spin-Off of the University of Florence, Via Santo Spirito,14-50125 Florence, Italy

**Keywords:** indigenous starter yeast, starter culture selection, 2D-PAGE, sulfur metabolism, hydrogen sulfide, glycerol, carbon partitioning, sensory analysis, heat shock proteins

## Abstract

Indigenous *Saccharomyces cerevisiae* strains persist within individual cellars and contribute to the character of spontaneously fermented wines, yet the molecular basis of their oenological differences remains largely unknown. In this work, eight predominant strains isolated from spontaneous fermentations in four Tuscan wineries were characterized by technological traits of oenological relevance and laboratory-scale fermentations of both a synthetic must and Sangiovese grape must. In each must, the strains reached similar final ethanol concentrations, although lag phase and maximum fermentation rate differed significantly, whereas the resulting wines differed significantly in glycerol, acetic acid and phenolic alcohol content and in the scores assigned by a trained sensory panel. Four strains spanning the observed range of behaviors were hence analyzed by two-dimensional polyacrylamide gel electrophoresis (2D-PAGE) and Matrix-Assisted Laser Desorption/Ionization Time-Of-Flight Mass Spectrometry (MALDI-ToF MS) at an early and a mid-fermentation stage, yielding 30–46 differentially abundant proteins identified per strain. Strain-specific abundance of proteins involved in glycerol formation, acetaldehyde oxidation and sulfur amino acid metabolism paralleled the corresponding differences in glycerol, acetic acid and hydrogen sulfide production, whereas glycolytic enzyme abundance varied without any corresponding variation in ethanol. Proteomic analysis thus identified candidate molecular correlates of the traits that distinguish strains, suggesting that it could complement traditional screening in the selection of indigenous starter cultures.

## 1. Introduction

The yeast *Saccharomyces cerevisiae* is the main microorganism responsible for alcoholic fermentation in winemaking, converting grape sugars into ethanol and carbon dioxide while simultaneously producing a complex set of primary and secondary metabolites that shape wine aroma, flavor, and mouthfeel. The biochemical processes associated with alcoholic fermentation reflect conserved stress-response programs that determine fermentation robustness under the physicochemical constraints of grape must (including, but not limited to, osmotic stress, low pH, ethanol production, and nutrient limitation), making *S. cerevisiae* both a fermentative workhorse and a major determinant of wine style [1,2,3].

*S. cerevisiae* is characterized by considerable intraspecific biodiversity, so that different strains display distinct fermentation performances and metabolic properties that influence the sensory profile of wine [4,5]. Native, or indigenous, *S. cerevisiae* strains involved in spontaneous fermentations confer particular organoleptic signatures on the wines of a given site, contributing to the concept of “microbial terroir” [4,6,7,8,9], and comparative studies have shown that wines fermented with indigenous strains differ in metabolite composition from those produced with commercial starters [10,11]. This could be specifically connected to strain-specific physicochemical and volatile profiles, as shown by some *S. cerevisiae* strains producing wines richer in desirable aroma compounds than those obtained with commercial cultures, thereby contributing to wine regionality [12].

Since consistent and predictable fermentations are usually obtained by inoculating grape must with selected starter cultures [2], the possibility of developing tailored starters from indigenous strains is of increasing interest in modern oenology. Such strains persist across vintages within the same cellar (the so-called “winery effect”) and display genetic, phenotypic and metabolic properties that distinguish them from commercial cultures [4,9,13,14,15,16]. Their selection typically relies on polyphasic approaches combining genotypic and phenotypic analyses to evaluate technological and oenological properties, such as stress tolerance, hydrogen sulfide production, killer phenotype, fermentation performance and the aroma and sensory profile of the resulting wines [17]. Recent works have additionally shown that proteomic analysis can resolve strain-specific physiological traits, including variation in stress-response and aroma-related proteins that points to diverse adaptive strategies during fermentation [13,18], and mass spectrometry-based approaches have been used to relate proteomic profiles to the oenological potential of wine yeasts [19,20]. Interestingly, whole-cell MALDI-ToF MS profiles of indigenous isolates have also been shown to reflect the oenological application of commercial strains, offering a rapid and cost-effective means of pre-selecting candidate starters suitable for preserving the distinctive character of wines from specific oenological areas [21]. Proteomics may therefore complement traditional selection methods in exploring the complex network of relationships that drive oenological fermentations.

The composition of grape must is itself a critical variable in comparative enological studies. As a complex matrix, differing widely in sugar composition, yeast-assimilable nitrogen sources, organic acids, micronutrients and phenolic content, grape must modulates yeast physiology [2]. Fully defined synthetic media that reproduce the key compositional features of grape juice are therefore commonly used to maintain experimental control, enabling reproducible comparisons and allowing differences to be interpreted as strain-derived rather than matrix-driven [22,23,24,25]. Despite growing interest, comprehensive characterization of indigenous *S. cerevisiae* strains under standardized fermentation conditions remains limited, and studies that combine oenological screening with molecular analysis of the same strains are scarce.

The present study accordingly combines an oenological and a proteomic characterization of indigenous *S. cerevisiae* strains isolated from naturally fermenting musts sampled in four wineries of four important Tuscan viticultural areas (Chianti, Chianti Classico, Montalcino and the central coastal area). Technological and oenological properties of selected yeast strains were first evaluated by in vitro tests and by laboratory-scale fermentations in both a synthetic and a Sangiovese grape must, and the resulting experimental wines were assessed by a trained sensory panel. Four strains, selected as spanning the range of oenological behaviors observed, were then characterized by two-dimensional polyacrylamide gel electrophoresis (2D-PAGE) and MALDI-ToF mass spectrometry at an early and a mid-stage of fermentation in the same synthetic must, with particular attention to proteins involved in the response to fermentation-related stresses and in the biochemical pathways underlying wine quality. By interpreting the proteomic data in the light of the oenological and sensory characterization of the wines produced by these strains, this integrated approach aims to identify candidate molecular correlates of the traits on which indigenous strains differ. Since such strains persist within individual cellars, the workflow described here could represent a potential route to identifying candidate starters among the strains predominating in each cellar. Information on the molecular background of strain behavior, used along with performance data obtained under defined conditions, could thus provide a promising additional criterion for the selection of indigenous starter cultures.

## 2. Results

### 2.1. Isolation and Technological Characterization of Indigenous S. cerevisiae Strains

To recover indigenous *S. cerevisiae* strains, spontaneous wine fermentations were monitored in four Tuscan wineries where commercial yeast strains were not used. Identification by RFLP-ITS PCR and typing by inter-delta sequence amplification showed that each fermentation was carried out by two predominant *S. cerevisiae* strains, each occurring at a frequency higher than 10% (Table 1).

**Figure 1:**
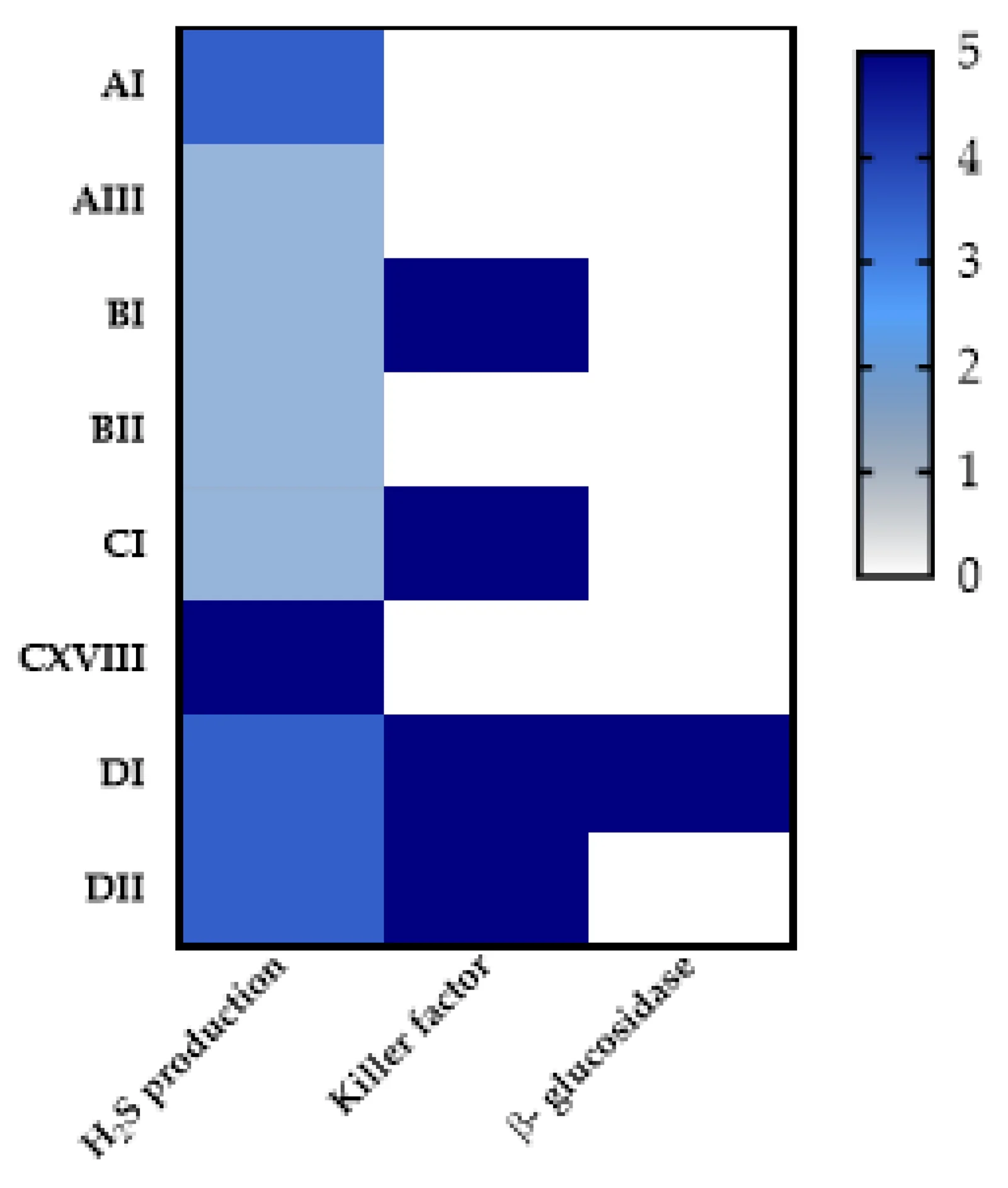
Heatmap of the technological traits of the eight indigenous *S. cerevisiae* strains determined by in vitro assays. Hydrogen sulfide production was graded on a six-point scale according to colony blackening on Bismuth Glucose Glycine Yeast Agar (BIGGY agar) (0 = white, 1 = beige, 2 = light brown, 3 = brown, 4 = dark brown, 5 = black). Β-Glucosidase activity and killer factor were scored as present (5) or absent (0).

The eight predominant strains were then characterized for three technological traits of oenological relevance: killer activity, which increases the likelihood of successful implantation in the must; β-glucosidase activity, which promotes the release of varietal terpenes from their glycosidic precursors; and hydrogen sulfide production, which can affect wine aroma. Killer activity and β-glucosidase activity were scored as present or absent. Four of the eight strains displayed the killer phenotype, and β-glucosidase activity was detected in strain D_I. Hydrogen sulfide production varied across the panel: strains A_III, B_I, B_II and C_I were low producers, strains A_I, D_I and D_II medium producers, and C_XVIII was the only high producer (Figure 1).

### 2.2. Fermentation Kinetics and Wine Composition in Synthetic Must 

The fermentative and chemical properties of the eight indigenous strains were first evaluated in laboratory-scale fermentations of the synthetic must SM2, a medium derived from that of Henschke and Jiranek [26], and adopted by the International Organisation of Vine and Wine (OIV) for the characterization of wine yeasts [25].

Fermentation progress was followed daily by weight loss until the weight remained constant for three consecutive days (Figure S1). Weight loss, expressed as CO_2_ produced per 100 mL of must, was fitted to a modified Gompertz function [27] to estimate the duration of the lag phase (λ), the maximum fermentation rate (μ_max_) and the maximum cumulative CO_2_ production I. R^2^ values ranged from 0.981 to 0.994, indicating a good fit in all cases (Table 2).

The two strains isolated from the same winery did not differ significantly from each other in either lag phase, μ_max_ or C (Table 2). Strains B_I and B_II, both isolated from winery B, fermented significantly faster than the strains from the other three wineries (μ_max_ 0.088 and 0.101 versus 0.053–0.064 dCO_2_/100 mL/h) without a corresponding gain in total CO_2_: B_I produced significantly less than every other strain (7.3 CO_2_/100 mL), whereas B_II (8.7) did not differ from the remainder (8.6–10.1).

At the end of fermentation, residual sugars, glycerol and other polyols, organic acids and phenolic alcohols were quantified (Table 3). Glucose was almost completely consumed by all strains (≤0.2 g/L), so residual sugar consisted essentially of fructose, and significant strain-dependent differences were found in fructose consumption and in the production of ethanol and secondary metabolites. Strain B_I left the highest residual fructose (6.9 g/L), followed by D_I (4.5 g/L) and C_XVIII (3.6 g/L), whereas B_II, C_I and D_II left less than 1 g/L and reached the highest ethanol concentrations (13.4% *v*/*v*, against 12.9% *v*/*v* for B_I). Among the polyols, C_XVIII produced significantly more glycerol than any other strain (5.8 versus 4.1–4.5 g/L), together with the highest concentrations of both the meso (0.19 g/L) and the racemic (0.58 g/L) forms of 2,3-butanediol. No significant differences were found for 1,2-propanediol. Acetic acid concentrations, by contrast, varied significantly: C_I and C_XVIII accumulated the highest levels (0.75 and 0.78 g/L), above the 0.56–0.69 g/L measured for the remaining six strains. Among the phenolic alcohols, tyrosol varied significantly across the panel, from 4.00 mg/L in A_III to 9.71 mg/L in C_XVIII. Hydroxytyrosol and tryptophol spanned narrower ranges (0.76–1.72 and 0.62–1.11 mg/L, respectively) with no significant differences between strains.

To quantify the overall magnitude of the difference among strains for each metabolite, global effect size was evaluated using eta-squared (η^2^), calculated via one-way Analysis of Variance (ANOVA) (Table S1). H^2^ represents the proportion of total variance in a given parameter that is attributable to the strain factor. Values of η^2^ were well above the conventional threshold for a large effect (η^2^ ≥ 0.14) [28] for most parameters, and reached or exceeded 0.70 for nine of the twelve parameters measured, indicating that strain identity accounted for at least 70% of the total variance in these cases, as against the differences between replicates reported in Table S1. The effect was strongest for racemic 2,3-butanediol (0.988), meso 2,3-butanediol (0.961), tyrosol (0.929), fructose (0.921) and acetic acid (0.915).

### 2.3. Fermentation Kinetics, Wine Composition and Sensory Profile in Grape Must

The same eight indigenous *S. cerevisiae* strains were then inoculated into 1 L of Sangiovese grape juice to assess their oenological behavior in a natural matrix. Fermentation was followed by daily weight loss and the data fitted to the modified Gompertz function [27] (Figure S2 and Table 4). R^2^ values ranged from 0.985 to 0.996, indicating a good fit throughout.

Lag phases were longer and more variable than in SM2, spanning 11 h (B_II) to 25 h (C_I), with the two strains isolated from winery C showing the longest lag phases (24 and 25 h). D_I and D_II reached the highest maximum fermentation rates (0.115 and 0.113 dCO_2_/100 mL/h), followed by C_I, C_XVIII and B_II (0.102–0.110) and by B_I (0.095), while A_I and A_III were the slowest (0.082 and 0.083). These differences in onset and rate did not translate into differences in total CO_2_ production, which was similar across the panel (9.2–9.8 CO_2_/100 mL) and in agreement with the chemical analysis of the resulting wines, which showed total residual sugars < 2 g/L and similar ethanol concentrations (13.00–13.35% *v*/*v*) (Table 5) for all strains.

Strain C_XVIII behaved in grape must similarly to what was observed in SM2, accumulating secondary metabolites at significantly higher levels than the other strains. It produced the highest concentrations of glycerol, of both 2,3-butanediol isomers, of 1,2-propanediol and of acetic acid, the last of these (0.45 g/L) nonetheless remaining below the threshold associated with sensory defects. Among the phenolic alcohols, hydroxytyrosol varied from 3.13 mg/L (A_III wine) to 4.73 mg/L (D_II wine), with B_II, D_I and D_II showing the highest values. Tyrosol showed the widest strain-dependent variation, from 16.9 mg/L (B_I) to 42.9 mg/L (D_II), whereas tryptophol ranged from 1.79 (A_III) to 3.54 mg/L (D_I) (Table 5).

Global effect size was also evaluated for the data presented in Table 5 (Table S2) [28]. Values of η^2^ ≥ 0.70 were obtained for eleven of the twelve parameters, and exceeded 0.90 for glycerol (0.929), meso (0.969) and racemic (0.974) 2,3-butanediol, acetic acid (0.926), tyrosol (0.968) and tryptophol (0.961), corresponding to more than 90% of the total variance in these parameters being attributable to the strain factor.

These experimental wines were submitted to sensory analysis: olfactory frankness, intensity and quality, and gustatory frankness, intensity, persistence and quality were scored, together with an overall impression in accordance with the RESOLUTION OIV/CONCOURS 332A/2009 [29] (Figure S3). Among the olfactory descriptors, the wines from A_I, A_III and D_I reached the highest intensity scores (6.1–6.3) and the highest olfactory quality, with A_I ranking first (13.3) ahead of A_III and D_I (12.6 each). The C_XVIII wine scored lowest on both frankness (4.0) and intensity (3.4), and correspondingly lowest on olfactory quality (9.4). A_I and A_III wines again scored highest for gustatory quality (17.3 and 16.9), combining high frankness with high persistence. The D_II wine reached the highest persistence score of the panel (7.1) but the lowest gustatory frankness (4.0), and its overall gustatory quality was accordingly low (13.7); the C_XVIII wine scored the lowest (13.4). The same ranking emerged in the general impression: A_III was the most appreciated wine (9.6), followed by A_I and B_I (9.1 each), while C_XVIII was the least appreciated (7.4).

### 2.4. Multivariate Statistical Analysis

**Figure 2:**
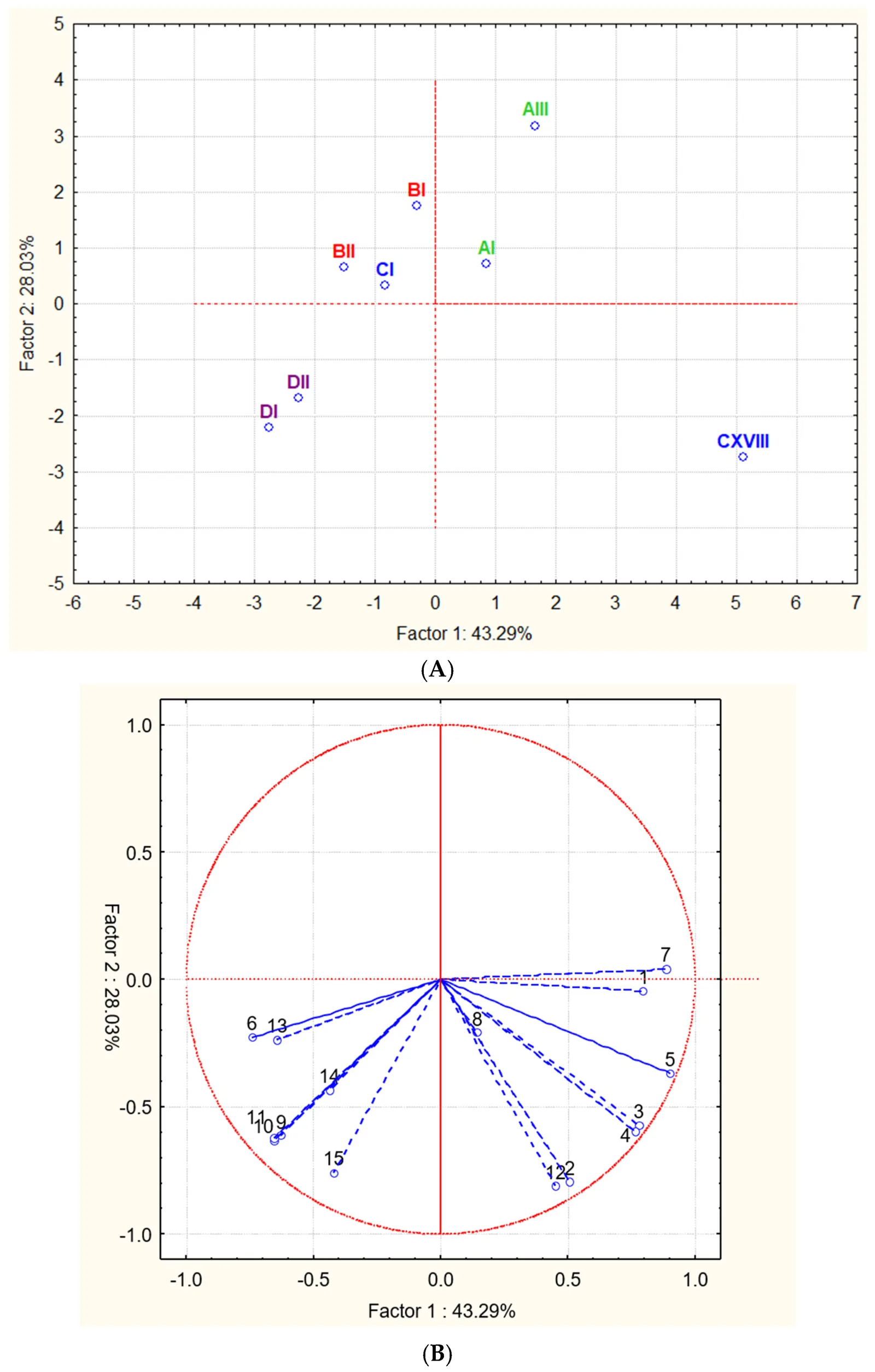
Exploratory Principal Component analysis (PCA) based on the results of in vitro assays and on chemical analysis of experimental wines obtained from grape must fermentations by indigenous *S. cerevisiae* strains. (**A**): similarity map determined by Principal Component (Factor) 1 and 2; (**B**): projection of the variables on the factor plane. Variables: 1, Fructose; 2, glycerol; 3, meso 2,3-butanediol, 4, racemic 2,3-butanediol (g/L); 5, 1,2 propanediol; 6, succinic acid; 7, acetic acid; 8, lactic acid; 9, hydroxytyrosol; 10, tyrosol; 11, tryptophol; 12, H_2_S production; 13, Killer factor; 14, β-glucosidase production; 15, maximum fermentation rate.

To explore the relationships between the oenological performance of the eight indigenous strains and the composition of the resulting wines, a Principal Component Analysis (PCA) was carried out combining the technological properties of the strains: killer factor, β-glucosidase and H_2_S production (Figure 1) with the maximum fermentation rate in grape must and the chemical composition of the corresponding Sangiovese wines (Figure 2). With eight strains and fifteen variables, the analysis is unsupervised and descriptive: it summarizes the structure of the dataset and does not support statistical inference. The first two components accounted for 71.32% of the total variance (PC1 = 43.29%, PC2 = 28.03%). C_XVIII was clearly separated from all other strains along PC1, consistent with the distinctive metabolic and fermentative profile described above. A_I and A_III clustered together in the quadrant defined by positive PC1 and positive PC2 scores, consistent with broadly similar overall characteristics. B_I, B_II and C_I grouped on the negative side of PC1, while D_I and D_II formed a separate cluster in the lower-left quadrant, separating them from the remaining strains along PC2.

**Figure 3:**
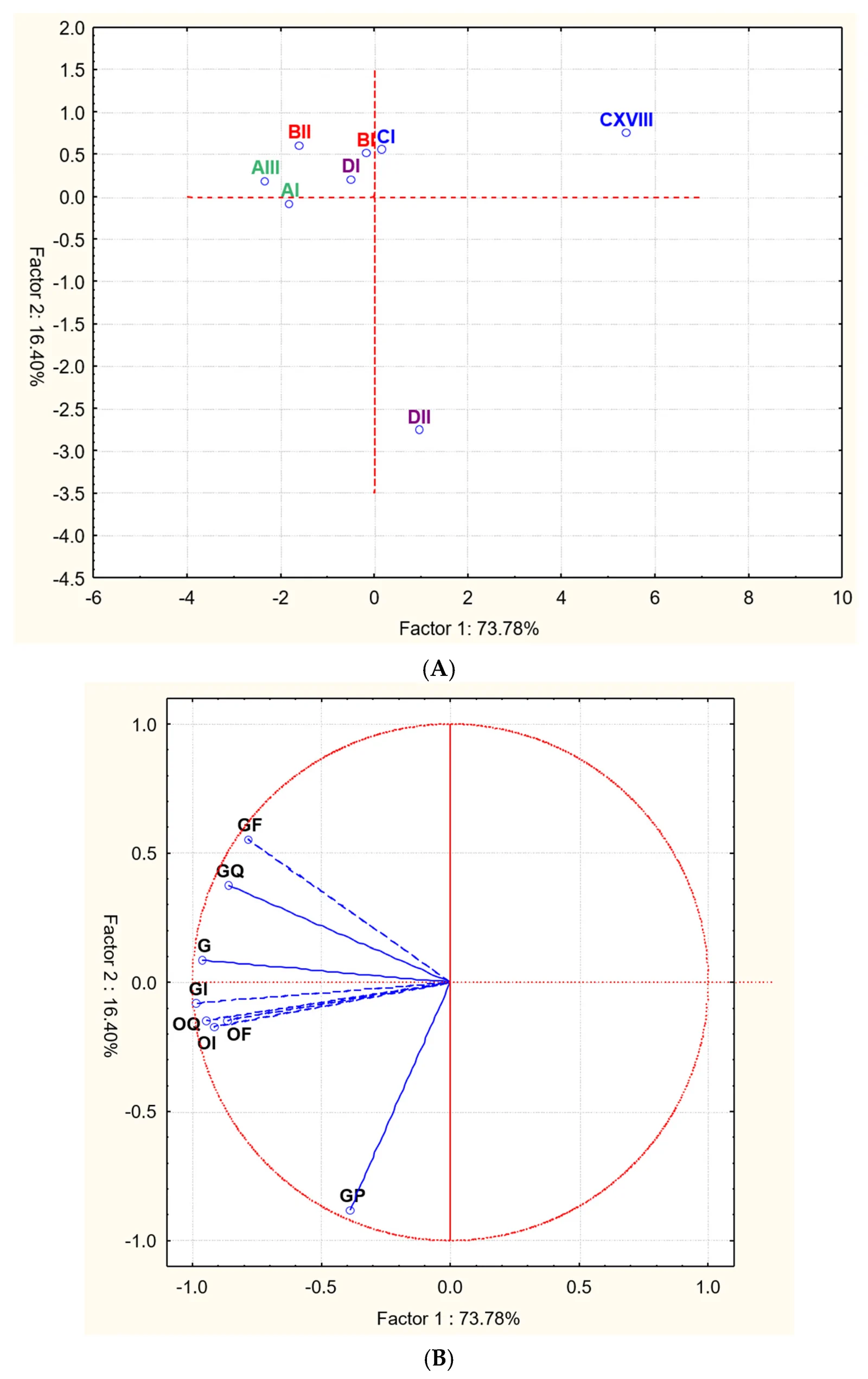
Exploratory Principal Component analysis (PCA) based on the sensory analysis of experimental wines obtained from grape must fermentations by indigenous *S. cerevisiae* strains. (**A**): similarity map determined by Principal Component (Factor) 1 and 2; (**B**): projection of the variables on the factor plane. Variables: OF: olfactory frankness; OI: olfactory intensity; OQ: olfactory quality; GF: gustatory frankness; GI: gustatory intensity; GQ: gustatory quality; GP: gustatory persistence; G: general impression.

A second PCA was performed on the sensory attributes of the same wines. Here, the first two components accounted for 90.18% of the total variance (PC1 = 73.78%, PC2 = 16.40%) (Figure 3A). The score plot again separated C_XVIII from the remaining strains along PC1, indicating a sensory profile distinct from that of the rest of the panel. A_I, A_III, B_I, B_II, C_I and D_I clustered closely together, whereas D_II was displaced along the negative direction of PC2, a separation driven mainly by gustatory persistence and, to a lesser extent, by gustatory frankness (Figure 3B).

Taken together, these two exploratory analyses indicate that the eight strains are distinguished along both their oenological and their sensory profiles, and that C_XVIII separates from the rest in both. On this basis, four strains spanning the range of observed behaviors, namely A_I, B_I, C_XVIII and D_II, were selected for proteomic characterization, with the aim of identifying candidate molecular correlates of the oenological and sensory traits on which these strains differed.

### 2.5. Comparative Proteomics

To characterize the molecular adaptations occurring during fermentation, the proteomes of strains A_I, B_I, C_XVIII and D_II were compared at two stages (early, 40 h, and mid, 76 h) of fermentation in synthetic must SM2. These analyses were carried out in an independent experiment in which all four strains were grown and sampled in parallel (Figure S4). Two-way repeated-measures ANOVA on log_10_-transformed viable counts showed a dominant effect of time (86.8% of total variation; F (2.72, 21.77) = 228.8, *p* < 0.0001), together with smaller but significant effects of strain (6.9%; F(3, 8) = 29.58, *p* = 0.0001) and of the time × strain interaction (2.6%; F(15, 40) = 2.329, *p* = 0.0168). Pairwise comparisons at individual timepoints, however, resolved only one significant difference over the whole time course, between B_I and C_XVIII at 88 h (*p* = 0.0220). The four strains therefore followed closely comparable growth kinetics and did not differ significantly at either of the two sampling points used for proteomic analysis (Figure S4).

We used a classical proteomic workflow combining 2D-PAGE with gel matching and MALDI-ToF MS for protein identification. About 1200 protein spots were detected in each 2D gel, and proteome map analysis enabled the unambiguous identification of 85 proteins in A_I, 93 proteins in B_I, 92 proteins in C_XVIII, and 87 proteins in D_II. To highlight differentially abundant proteins (DAPs), relative spot % volume ratios (mid- vs. early time-points) were calculated and those exhibiting a fold-change (FC) ≥ 2.0 (increased abundance) or ≤ 0.5 (decreased abundance) were considered (Figure S5 and Table 6). Annotated 2D gels are available in Figure S6. The number of DAPs varied among strains, with B_I showing the highest number of changes (46 DAPs), followed by D_II (36 DAPs), and A_I and C_XVIII (30 DAPs) (Figure S5).

Chemical analysis of the fermenting must confirmed that the two proteomic sampling points corresponded to an active fermentation phase where viable counts were increasing (Figure S4), hexose were consumed and ethanol was produced. The sampling point at 40 h was in the transition time where hexose consumption was shifting from ≈ 13–14% (measured at 28 h) to ≈19-≈29% (measured at 52 h) of the initial hexoses (230 g/L) with ethanol rising from 0.3–1.3 g/L to 2.7–6.7 g/L over the same interval. At the mid sampling point (76 h), consumption had risen to ≈ 37–40% in all four strains and ethanol had reached 6.7–15.0 g/L.

**Figure 4:**
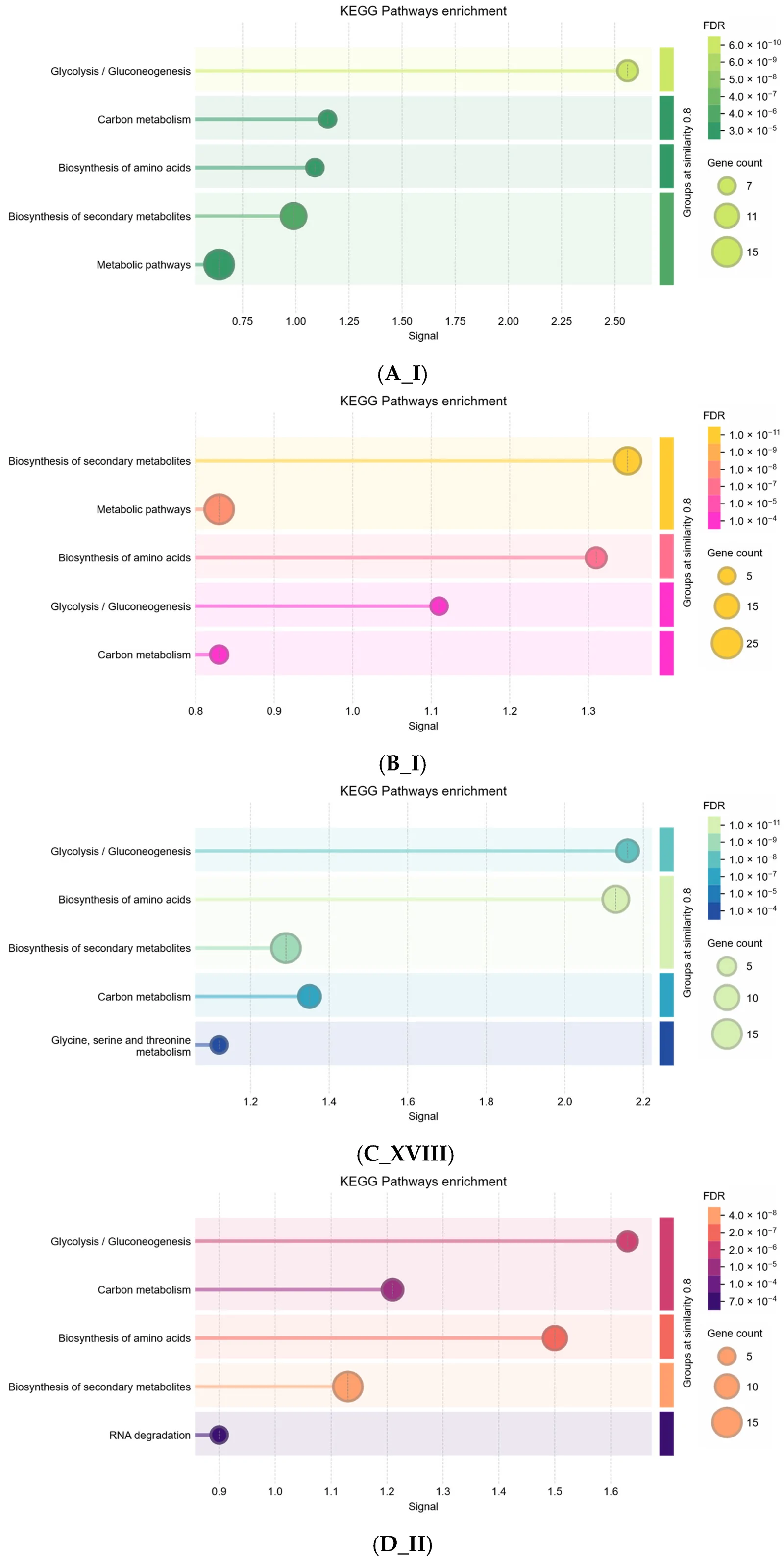
KEGG pathway enrichment among the differentially abundant proteins of strains (DAPs) (**A_I**, **B_I**, **C_XVIII**, **D_II**) comparing mid (76 h) to early (40 h) fermentation stage in synthetic must SM2. Enrichment was computed with STRING v12.0 against the whole *S. cerevisiae* proteome; the five terms with the highest signal are shown for each strain. Signal is a weighted harmonic mean of the observed/expected ratio and −log(False Discovery Rate, FDR). Dot size indicates the number of DAPs annotated with each term and dot color the FDR (Benjamini–Hochberg). The colored band at the right groups terms clustered at 0.8 functional similarity.

Functional annotation of the DAPs returned the same core categories in every strain: glycolysis and gluconeogenesis, carbon metabolism, and the biosynthesis of amino acids and secondary metabolites (Figure 4; Tables S3–S6). A smaller number of strain-specific categories were also annotated, including RNA degradation in B_I and D_II, cysteine and methionine metabolism in C_XVIII, and, in D_II, three vacuolar H^+^-ATPase subunits (annotated to the KEGG term ‘phagosome’) (Figure 4; Tables S3–S6). Coomassie-stained 2D maps preferentially resolve abundant proteins, and enrichment was computed against the whole proteome as background; the categories recurring in all four strains are therefore those expected for the most abundant fraction of the proteome. Enrichment results are accordingly reported as a descriptive summary of the functions represented among the DAPs, since enrichment of proteins annotated to a pathway identifies the functional categories present in the input list and does not by itself demonstrate that the pathway is regulated.

#### 2.5.1. Central Carbon Metabolism

Glycolytic enzymes displayed divergent abundance patterns across strains. Enolase 1 (ENO1) was strongly increased in abundance in strains A_I (FC 2.4) and D_II (FC 3.0), while pyruvate kinase (KPYK1) appeared only at mid fermentation in C_XVIII and was increased in abundance in D_II (FC 2.4). Glyceraldehyde-3-phosphate dehydrogenase isoforms differed markedly between strains: A_I showed decreased abundance of G3P1 (FC 0.4) and G3P3 (FC 0.3), and B_I showed increased abundance of G3P3 (FC 2.1), and in C_XVIII G3P1 was detected only at the mid-fermentation stage. Hexokinase-2 (HXKB) was increased in abundance in A_I (FC 2.0) and decreased in abundance in D_II (FC 0.5). Strain B_I showed increased abundance of transaldolase (TAL1, FC 7.3).

Pyruvate decarboxylase 1 (PDC1) showed a negative trend in all four strains, with a FC 0.4 in strain A_I. The mitochondrial pyruvate dehydrogenase E1 component (ODPB) was increased in abundance in A_I (FC 2.3) and B_I (FC 2.5) but decreased in abundance in D_II (FC 0.2). Residual pyruvate in the medium was closely comparable across the four strains at the sampling times flanking the proteomic time points (0.02–0.03 g/L at 28 h, 0.09–0.11 g/L at 52 h and 0.11–0.13 g/L at 76 h) (Table S7).

#### 2.5.2. Amino Acid Metabolism and Aroma Precursors

Interesting strain-specific differences were observed in amino acid biosynthesis pathways. Tryptophan synthase (TRP) was increased in abundance in D_II (FC 5.8), B_I (FC 2.9), and A_I (FC 2.2) strain. 3-isopropylmalate dehydrogenase (LEU3) was increased in abundance in D_II (FC 2.1). ATP phosphoribosyltransferase (HIS1) was decreased in abundance in A_I (FC 0.3) and D_II (FC 0.4).

Sulfur amino acid metabolism showed notable regulation in strain C_XVIII, where homocysteine synthase (CYSD, FC 2.0) and cystathionine β-synthase (CBS, FC 2.6) were increased in abundance, while cystathionine γ-lyase (CYS3, FC 0.5) was decreased in abundance; CBS and CYS3 were detected in this strain only. In strain B_I, both S-adenosylmethionine synthases were decreased in abundance (METK1, FC 0.3; METK2, FC 0.4).

Enzymes related to sterol and isoprenoid biosynthesis also differed among strains. Farnesyl pyrophosphate synthase (ERG20) was increased in abundance in B_I (FC 2.5) and appeared only at mid-fermentation stages in D_II, while A_I and C_XVIII showed no changes. Similarly, Sterol 24-C-methyltransferase (ERG6) appeared only at mid fermentation stages in strain B_I.

#### 2.5.3. Stress Response Proteins

Heat shock proteins and chaperones exhibited strain-specific abundance patterns. Heat shock protein 60 (HSP60) was strongly increased in abundance in A_I (FC 5.7) but decreased in abundance in B_I (FC 0.4), C_XVIII (FC 0.5), and D_II (FC 0.2). Heat shock protein SSE1 (HSP7F) was increased in abundance in A_I (FC 2.0) and C_XVIII (FC 2.2) but decreased in abundance in B_I (FC 0.4) and D_II (FC 0.3). The 12 kDa heat shock protein (HSP12) showed a negative trend in all strains, being decreased in abundance in B_I (FC 0.4), C_XVIII (FC 0.5) and D_II (FC 0.5), similarly to the endoplasmic reticulum chaperone BiP (BIP), which was decreased in abundance in B_I (FC 0.2) and C_XVIII (disappearing at mid fermentation stage).

Aldehyde dehydrogenase 2 (ALDH2) was increased in abundance in C_XVIII (FC 5.8) and A_I (FC 2.0), compatible with a higher capacity for acetaldehyde oxidation in these strains.

#### 2.5.4. Other Metabolic Functions

Further noteworthy changes included differential abundance of ribosomal proteins, with several proteins showing strain-specific patterns suggesting altered translational capacity. Proteins involved in nucleotide metabolism, such as adenosine kinase (ADK, decreased in abundance in A_I, FC 0.3) and CTP synthase 1 (URA7, increased in abundance in D_II, FC 4.0), also showed variation among strains.

## 3. Discussion

This study combined an oenological and a proteomic characterization of indigenous *S. cerevisiae* strains isolated from spontaneous fermentations in four Tuscan wineries located in four important viticultural areas. The two predominant strains from each winery were screened for technological traits and for their fermentative behavior in both a fully synthetic must adopted by the OIV for the characterization of wine yeasts and a Sangiovese grape must, and the resulting wines were assessed by a trained panel. Four strains spanning the range of observed behaviors were then examined by 2D-PAGE and MALDI-ToF MS at an early and mid-stage of fermentation. This combination made it possible to identify strain-specific differences in protein abundance that represent candidate molecular correlates of the oenological and sensory traits on which the strains differed.

### 3.1. Proteome Remodeling During Active Fermentation

All four selected strains reorganized their proteome substantially between the early and the mid-stage of fermentation, with 30 to 46 DAPs identified per strain. Functional annotation of these proteins returned the same core categories in every strain (glycolysis and gluconeogenesis, carbon metabolism, and the biosynthesis of amino acids and secondary metabolites) together with a smaller number of strain-specific categories, including RNA degradation in B_I and D_II, cysteine and methionine metabolism in C_XVIII and vacuolar H^+^-ATPase subunits in D_II. Since enrichment was computed against the whole proteome, and since Coomassie-stained 2D gels preferentially resolve the abundant fraction of the proteome, these enrichment results are best read as a descriptive summary of the functions represented among the identified DAPs rather than as evidence that these pathways were selectively regulated.

The extent of remodeling varied among strains, but this seemed unrelated to fermentation quality: B_I, the most extensively remodeled strain, was also the one that fermented least completely in synthetic must, whereas A_I and C_XVIII, with the fewest changes, produced, respectively, one of the highest (A_I general impression 9.1) and the lowest (C_XVIII general impression 7.4) scoring wine of the four (Figure S3). Strain B_I, however, also displayed the highest maximum fermentation rate of the four strains in synthetic must (Table 2), raising the possibility that rapid fermentation might imply a greater requirement for proteome reorganization.

### 3.2. Proteomic Signatures and Their Oenological Correlates

**Figure 5:**
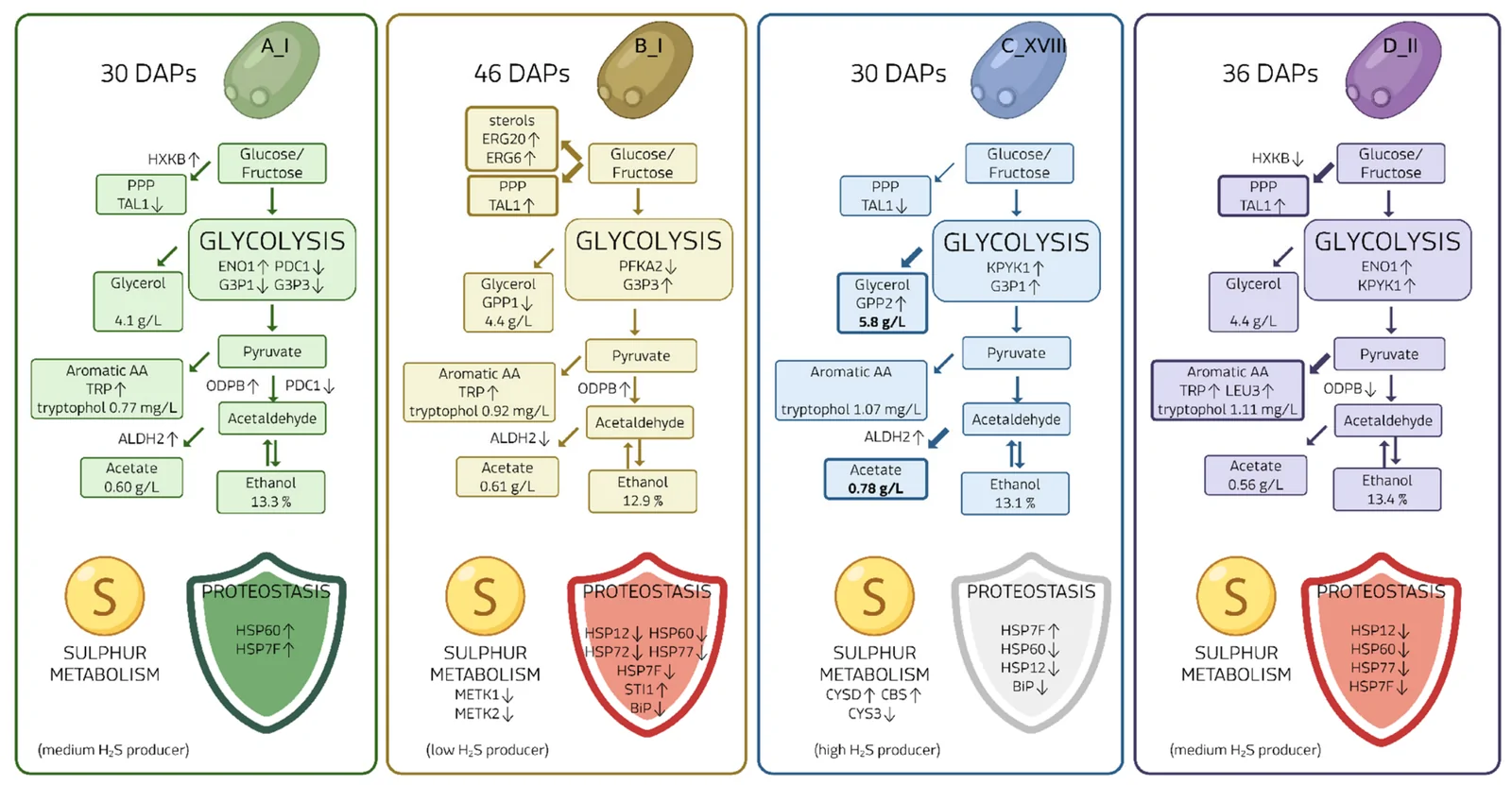
Protein abundance changes and associated end-product concentrations in the four indigenous *S. cerevisiae* strains characterized at the proteome level. For each strain, the enzymes whose abundance varied between the mid (76 h) and the early (40 h) fermentation stage in synthetic must SM2 are positioned at the metabolic step they catalyze, (↑, fold-change ≥ 2.0 or protein detected only at mid stage; ↓, fold-change ≤ 0.5 or protein absent at mid stage), with the total number of differentially abundant proteins (DAPs) given for each strain. Metabolite concentrations are those measured at the end of fermentation in the same medium (SM2, Table 3), shown in bold where the strain differed significantly from the others. Arrow thickness is scaled to end-product concentrations, the arrows to ethanol being of equal weight in all panels as ethanol production did not differ appreciably among strains. Shields summarize the chaperone complement (green, predominantly increased; red, predominantly decreased; grey, changes in both directions), and sulfur amino acid metabolism is annotated with the H_2_S phenotype determined on BIGGY agar (Figure 1). PPP, pentose phosphate pathway; AA, amino acids.

The proteomic characterization was performed in a separate experiment designed to resolve two stages of active fermentation, and the proteomic features of each strain are therefore discussed below in relation to its oenological phenotype, bearing in mind that the proteomic experiment was conducted in synthetic must only. Ethanol production differentiated the four strains only marginally, varying by no more than 0.5% *v*/*v* in synthetic must (Table 3) and not differing significantly in Sangiovese must (Table 5); the discriminating differences concerned the partitioning of carbon towards secondary products and the technological traits underlying the sensory outcome (Figure 5). All the relationships described below are associations between protein abundance and oenological phenotype across four strains; the experimental design adopted here does not allow causal inference, and the proposed mechanisms are interpretations consistent with established biochemistry rather than demonstrated causes.

#### 3.2.1. Strain A_I: Increased Chaperone Abundance and the Soundest Wine of the Panel

A_I was the only strain to show increased abundance of heat shock protein 60 (FC 5.7) and one of two to show increased abundance of SSE1 (FC 2.0), against a general reduction in chaperone abundance in the remaining strains. A_I fermented synthetic must to 13.3% *v*/*v* ethanol with 2.3 g/L residual fructose and produced glycerol and acetate concentrations among the lowest of the panel, together with the highest olfactory (13.3) and gustatory (17.3) quality scores of the eight wines. Glycerol and acetic acid both accumulate when fermenting cells experience osmotic and redox imbalance, the former as the principal cytosolic sink for excess NADH and the latter through acetaldehyde oxidation, and their concentrations are commonly regarded as indicators of the physiological condition of the culture [30]. In A_I, the induction of HSP60 and SSE1 at a stage where the other three strains reduced their chaperone complement coincided with low glycerol and acetate concentrations in synthetic must and with the highest sensory scores of the evaluation panel. Increased chaperone abundance is consistent with effective management of proteotoxic stress [31,32] and provides a plausible molecular correlate of the overall soundness of this strain. Whether the maintenance of proteostasis contributes causally to this outcome cannot be established from the present data; it does, however, identify chaperone competence during fermentation as a candidate trait for strain selection, testable by comparing strains under defined stress conditions. Whether this advantage would be maintained under more demanding conditions (e.g., higher initial sugar, lower assimilable nitrogen, lower temperature) remains to be established by targeted stress-challenge fermentations.

#### 3.2.2. Strain B_I: Pentose Phosphate Diversion with Reduced Chaperone Capacity

Strain B_I combined the largest number of DAPs with a broad reduction in the chaperone complement: HSP60, SSA2, SSE1, HSP12, BiP and the mitochondrial import motor subunit (FC ≤ 0.5), the exceptions being peptidyl-prolyl cis-trans isomerase D (FC 5.9) and STI1 (detected only at the mid-fermentation stage). The same strain showed the poorest sugar utilization of the panel in synthetic must, leaving the highest residual fructose (6.9 g/L) and reaching the lowest ethanol concentration (12.9% *v*/*v*), while fermenting Sangiovese must to near-completion (0.45 g/L residual fructose). This matrix dependence suggests a conditional limitation, exposed by the more restrictive nutritional environment of the synthetic medium.

B_I also carried the largest single fold-change of the dataset, transaldolase (TAL1, FC 7.3), together with increased abundance of farnesyl pyrophosphate synthase (ERG20, FC 2.5), the appearance of sterol 24-C-methyltransferase (ERG6) at the mid-fermentation stage, and decreased abundance of both S-adenosylmethionine synthases (METK1, FC 0.3; METK2, FC 0.4). A higher transaldolase abundance is compatible with an increased flux through the pentose phosphate pathway, which would supply NADPH for reductive biosynthesis and ribose-5-phosphate for nucleotide synthesis and would support the sterol and isoprenoid biosynthesis indicated by the ERG20 and ERG6 changes, while diverting carbon from glycolysis. This is consistent with the incomplete sugar utilization observed in synthetic must, although pathway fluxes were not measured. Since farnesyl pyrophosphate is a precursor of isoprenoid compounds relevant to wine aroma [33,34], this reallocation may carry aromatic as well as fermentative consequences, which would require volatile analysis to establish.

#### 3.2.3. Strain C_XVIII: Carbon Diverted to Polyols and Acetate, with Dysregulated Sulfur Assimilation

C_XVIII separated from the remaining strains in both PCAs and presented the least favorable oenological profile: the sole high producer of H_2_S among the eight isolates, the highest concentrations of glycerol and of both 2,3-butanediol isomers in both fermentation matrices, and the lowest score on every sensory descriptor except gustatory frankness (general impression 7.4, against 9.6 for the highest-ranked wine) (Figure S3). Three features of its proteome correlate to these traits.

(i) Glycerol-1-phosphate phosphohydrolase 2 (GPP2) was increased in abundance in C_XVIII alone (FC 2.1) and unchanged in the other three strains, and C_XVIII produced the most glycerol in both synthetic must (5.8 against 4.1–4.5 g/L) and Sangiovese must (8.05 against 6.10–7.05 g/L). Glycerol formation through the Gpp route is the principal cytosolic sink for excess NADH under fermentative conditions and therefore competes with ethanol for carbon and reducing equivalents. This represents the closest correspondence between enzyme abundance and product accumulation observed in this study, also being confirmed in both fermentation matrices.
(ii) Aldehyde dehydrogenase (ALDH2) was increased in abundance 5.8-fold in C_XVIII, the largest change recorded for this enzyme, and among the four strains characterized C_XVIII accumulated the highest acetic acid concentrations in both fermentation matrices (0.78 g/L in SM2; 0.45 g/L in Sangiovese must). This association, however, seemed not to reflect a graded relationship, since acetic acid concentrations in the other three strains were comparable (0.56–0.61 g/L in SM2) irrespective of ALDH2 abundance. The concurrence in C_XVIII of the highest glycerol and acetic acid concentrations is also consistent with the metabolic coupling of these two by-products through the maintenance of redox balance, a co-variation already reported for other indigenous *S. cerevisiae* strains [35]. Acetic acid in the experimental Sangiovese wine nevertheless remained below the concentration associated with sensory defect, so that this trait differentiates the strain analytically without necessarily compromising wine quality.
(iii) C_XVIII displayed a distinctive reconfiguration of sulfur amino acid metabolism, with increased abundance of homocysteine/cysteine synthase (CYSD, FC 2.0) and cystathionine β-synthase (CBS, FC 2.6) alongside decreased abundance of cystathionine γ-lyase (CYS3, FC 0.5); CBS and CYS3 were detected in this strain only. Restriction of the CYS3 step while flux into cystathionine is maintained would be expected to limit cysteine formation, and a reduced cysteine pool would in turn relieve the feedback repression that restrains sulfate assimilation [36]. Sulfide produced in excess of the capacity for its incorporation into organic sulfur would then be released as H_2_S, which is consistent with the phenotype determined on BIGGY agar and with the low olfactory frankness (4.0) and intensity (3.4) recorded for the corresponding wine, although neither the cysteine pool nor the sulfate assimilation flux was measured here. A reduced cysteine pool would further constrain glutathione biosynthesis, with consequences for the antioxidant and redox buffering capacity of the cell [37,38].

In C_XVIII, an in vitro technological trait, a proteomic abundance pattern and a sensory outcome are therefore concordant. This concordance identifies a candidate molecular correlate of the oenological phenotype observed, but establishing a causal relationship would require targeted experiments outside the scope of the current exploratory framework.

#### 3.2.4. Strain D_II: Enhanced Aromatic Amino Acid Biosynthesis

D_II showed the strongest increase in the abundance of tryptophan synthase of any strain (TRP, FC 5.8), together with 3-isopropylmalate dehydrogenase (LEU3, FC 2.1), lysyl-tRNA ligase (FC 3.6) and CTP synthase (FC 4.0), suggesting a coordinated increase in biosynthetic capacity for aromatic and branched-chain amino acids and for nucleotides. Among the four strains characterized at the proteome level, D_II produced the most tryptophol in Sangiovese must (2.72 against 1.93–2.12 mg/L). Tryptophol derives from tryptophan through the Ehrlich pathway, so the correspondence between TRP abundance and tryptophol accumulation is biochemically plausible, although the present data do not establish it. This phenomenon was apparent in grape must but not in synthetic must, where tryptophol did not differ among strains. Tryptophan and leucine are also precursors of higher alcohols and their acetate esters, which contribute to aromatic complexity at moderate concentrations and become undesirable when they accumulate [39,40]. Notably, the production of Ehrlich-pathway alcohols, including leucine-derived isoamyl alcohol and phenylalanine-derived 2-phenylethanol, has been shown to be strain-dependent, supporting the discriminatory value of such analysis among indigenous *S. cerevisiae* isolates [41]. Since SM2 supplies only 26 mg/L tryptophan and 76 mg/L leucine, de novo biosynthesis rather than catabolism of exogenous amino acids is the more probable route under these conditions, and the potential for higher alcohol formation in nitrogen-richer grape musts may be correspondingly greater.

#### 3.2.5. Correspondence Between Enzyme Abundance and Metabolic Phenotype

The associations identified above involve exclusively pathways branching from central glycolytic metabolism: GPP2 with glycerol, ALDH2 with acetate, the sulfur assimilation module with H_2_S, and tryptophan synthase with tryptophol. No comparable correspondence was observed for glycolysis itself. Enolase 1 was increased in abundance in A_I (FC 2.4) and D_II (FC 3.0), hexokinase-2 in A_I alone (FC 2.0), and pyruvate kinase was detected only at the mid-fermentation stage in C_XVIII, yet final ethanol concentrations differed by no more than 0.5% *v*/*v* across the eight strains. An equivalent dissociation was apparent within the proteomic experiment, where pyruvate concentrations at the sampling times bracketing the proteomic analysis were closely comparable across strains despite a more than tenfold range in the abundance of the mitochondrial pyruvate dehydrogenase E1 component (FC 2.5 in B_I against 0.2 in D_II).

This dissociation is in agreement with the established finding that flux control in yeast glycolysis is distributed across the pathway, the flux control coefficient of any individual enzyme being low, so that its increased abundance does not in itself increase the rate of ethanol formation [42,43,44]. Enzymes at metabolic branch points are subject to different constraints, since they compete for a common intermediate pool and their relative abundance therefore exerts a more direct influence on the partitioning of carbon between alternative routes [45]. The pattern observed here, in which a conserved ethanol yield is accompanied by strain-specific accumulation of glycerol, acetate, sulfur compounds and phenolic alcohols, corresponds to that predicted by this asymmetry. A practical implication for strain selection is that proteomics may prove informative for the secondary metabolites that determine sensory quality.

### 3.3. Considerations on Experimental Design and Future Directions

The oenological and the proteomic analyses presented in this work were designed to address different questions and were conducted accordingly. The fermentation trials described in Section 2.2 and Section 2.3 were performed to reproduce the kinetics and the final composition obtained in practice, whereas the proteomic experiment was configured to resolve two defined stages of active fermentation, all four strains being grown and sampled in parallel so that the comparisons between strains and between stages are internally controlled. The two datasets are therefore complementary, and the correspondences described in Section 3.2 are established at the level of the strain rather than of matched fermentation conditions.

The composition of the two matrices itself could have also affected the proteome. SM2 supplies nitrogen as a defined mixture of amino acids plus 100 mg/L diammonium phosphate, whereas the Sangiovese must contained 120 mg/L ammonia and 30 mg/L α-amino nitrogen. SM2 also lacks the phenolic fraction, contains sterols and unsaturated-fatty-acid substitutes at fixed concentrations, was not sulfited, was adjusted to pH 3.3 against pH 3.6 in the grape must, and differs in the content of solids and micronutrients. Nitrogen source and availability, lipid and sterol supply, and oxidative or sulfite-related stress are all known modulators of the pathways examined here, including amino acid biosynthesis, sterol and isoprenoid synthesis, and the stress-response and sulfur assimilation machinery. The protein abundance data reported here therefore describe the behavior of the four strains in SM2, and their extrapolation to wines produced in grape must is correspondingly indirect.

Two observations within our dataset point in the same direction First, strain B_I showed the poorest sugar utilization of the panel in SM2, leaving 6.9 g/L residual fructose, yet fermented Sangiovese must almost to completion (0.45 g/L). Second, the strain difference in tryptophol was itself matrix-dependent: concentrations differed among strains in grape must, where D_II accumulated the most, but not in synthetic must. Since the proteome was analyzed in SM2 only, the correspondence described in Section 3.2.4 relates a protein abundance measured in synthetic must to a metabolite difference observed in grape must, an inference that the present design cannot test directly. Repeating the proteomic comparison in grape must, with proteomic and chemical sampling from the same vessels, is therefore the most informative next step, and would establish which of the abundance patterns described here persist in a natural matrix.

Protein extracts from independent fermentations were pooled for each strain and time point, so that each gel represents the average of biological replicates and the between-culture variability is integrated into a single map. This design favors the reliable detection of consistent changes over the estimation of variance, and DAPs were accordingly identified by fold-change thresholds. Another limitation concerns some visible streaking in the SDS-PAGE gels (Figure S6), which might indicate that protein extraction and/or solubilization were not fully optimal and may have affected spot resolution and protein identification. Both limitations would be addressed by extending coverage through shotgun LC-MS/MS [18,30], which would make regulatory, membrane and low-abundance proteins accessible and would establish whether the branch-point associations reported here are accompanied by changes in the corresponding regulatory networks.

The metabolite analyses were directed at the major fermentation products, and the volatile fraction was not examined. Profiling volatile sulfur compounds, higher alcohols and acetate esters would allow the interpretations advanced here to be tested for sulfur metabolism in C_XVIII, for isoprenoid biosynthesis in B_I and for aromatic amino acid metabolism in D_II and would establish whether these proteomic signatures translate into the aroma profiles they suggest. This is in line with comprehensive two-dimensional GC analyses that have proved suitable for discriminating indigenous strains by their higher alcohol, ester and volatile acid profiles [41].

Taken together, the combination of oenological screening with proteomic analysis proved informative for the characterization of indigenous wine yeasts, providing candidate molecular correlates of the traits by which the strains were effectively distinguished. C_XVIII received the lowest panel scores for olfactory frankness and intensity, and its proteome shows both the reconfiguration of sulfur amino acid metabolism underlying its H_2_S production and the GPP2 and ALDH2 changes associated with its high glycerol and acetate concentrations. A_I, by contrast, combined the highest sensory scores of the panel with glycerol and acetic acid concentrations among the lowest, and with the only sustained chaperone response among the four strains.

Proteomics may therefore occupy a defined position within a multi-step selection scheme, not as a substitute for fermentation trials and sensory evaluation, but as a means of characterizing the molecular background of a given strain behavior and hence making hypotheses about its performance under conditions not yet tested [46]. A strain excluded on the grounds of H_2_S production for one application may prove suitable where sulfur precursors are limited or where nitrogen supplementation is applied, the candidate mechanism being identified rather than the phenotype merely observed. B_I, whose limitation became evident only in the nutritionally restrictive synthetic medium, would warrant evaluation across musts of differing nitrogen status, while a strain showing a sustained chaperone response, as A_I does, would be a candidate for the more demanding fermentation conditions that a standard screening does not impose. Such information would be valuable to turn a phenotypic ranking into a set of testable predictions, but must be corroborated under cellar conditions, since the laboratory-scale performance of indigenous strains does not necessarily transfer to pilot-scale winery fermentations [35].

Each *S. cerevisiae* strain characterized in this work was isolated from a single winery, with the two strains associated with each winery being the two that predominated in that cellar. Therefore, strain and winery are completely confounded in our design, and no effect attributable to the winery as such can be separated from differences between strains. Indigenous *S. cerevisiae* populations have been reported to persist across vintages within individual cellars, a phenomenon referred to as the “winery effect” [9,47], but testing it would require several strains per winery across consecutive vintages, which was outside the scope of this work. What the present design establishes is that the strains differ from one another, and that each is associated with the cellar in which it predominated. Each of the strains characterized here would, if reintroduced as a starter culture in that cellar, operate under the conditions in which it was found to predominate, namely the same equipment, grape supply and resident microbiota. Whether this confers any advantage over strains from other cellars remains to be tested. The individual cellar is also the scale at which such results would be applied by a producer. The workflow adopted in the present study is transferable to that context in its entirety: the predominant isolates of a cellar are identified and screened for technological traits, their oenological behavior is assessed in laboratory-scale fermentations and by sensory evaluation, and the molecular basis of the traits discriminating among them is subsequently resolved by proteomic analysis. Whether comparable molecular signatures characterize a viticultural area as a whole, rather than the individual cellars within it, remains a distinct question, the resolution of which would require the analysis of several cellars over consecutive vintages.

## 4. Materials and Methods

### 4.1. Isolation of Indigenous Saccharomyces spp. Yeasts

Spontaneous fermentations were set up in four Tuscan wineries that do not use commercial yeast starters for their wine production. When two-thirds of the sugar was fermented, yeasts were isolated using plates of WL Nutrient Agar medium (Oxoid Ltd., Basingstoke, Hampshire, UK) containing sodium propionate (VWR International Srl, Milan, Italy) (2 g/L) and streptomycin (VWR International Srl, Milan, Italy) (30 mg/L) to inhibit the growth of molds and bacteria, respectively. Colonies showing color and morphology characteristic of the genus *Saccharomyces* (umbonate, creamy-white) were purified by subsequent streaking, grown on YPD medium (1% *w*/*v*), and maintained in a solution containing 50% (*v*/*v*) glycerol at −80 °C until use.

### 4.2. Identification and Typing of Saccharomyces cerevisiae Strains

Approximately 100 yeast isolates from each winery were identified as belonging to the *Saccharomyces cerevisiae* species by amplification of the 5.8S rRNA gene and of the two ribosomal internal transcribed spacers, by using the primer pair ITS1/ITS4 (Thermo Fisher Diagnostics S.p.A., Rodano, Milan, Italy). The obtained amplicons were digested using the restriction enzymes *Hae*III, *Cfo*I, *HinfI* and *Dde*I (Life Technologies Italia, Monza, Italy), as described by Esteve-Zarzoso et al., 1999 [48]. All these isolates were also typed by inter-δ PCR with δ12 / δ21 the primer pair (Thermo Fisher Diagnostics S.p.A., Rodano, Milan, Italy) as reported by Legras and Karst [49]. PCR reactions included both negative (DNA-free) and positive controls and were processed in an Applied Biosystems^®^ 2720 Thermal Cycler (Life Technologies, Monza, Italy). For each *S. cerevisiae* strain typed, the isolation frequencies were calculated, and two strains with the highest isolation frequencies were chosen for each winery.

### 4.3. Technological Characterization of S. cerevisiae Strains

Technological characteristics including killer character, capability to produce hydrogen sulfide and beta-glucosidase activity were investigated. Killer activity tests were performed according to Philliskirk and Young [50], on a medium (MYB) containing malt extract broth (2% *w*/*w*), methylene blue 0.003% *w*/*w* and agar (2% *w*/*w*), buffered at pH 4.76 with 0.1 M citric acid phosphate buffer [50]. The reference sensitive strain *S. cerevisiae* (NCYC 1006; National Collection of Yeast Cultures, Norwich, UK) and the killer reference strain *S. cerevisiae* NCYC 738 (National Collection of Yeast Cultures, Norwich, UK) were used. The tested isolates were designated as killer strains (K) when the spot was surrounded by a clear zone with a blue border in which no growth of the inoculated sensitive strain had occurred.

The capability to produce hydrogen sulfide was estimated as reported by Aponte et al. [51] by spotting 5 μL of a fresh yeast culture on Bismuth Glucose Glycine Yeast Agar (BIGGY) agar plates (Oxoid Ltd., Basingstoke, UK). Hydrogen sulfide (H_2_S) production was estimated by colony blackening after three days at 28 °C, using a six-point scale: 0 = white, 1 = beige, 2 = light brown, 3 = brown, 4 = dark brown, 5 = black.

Beta-glucosidase activity tests were performed according to Hernández et al. [52] on Agar esculin medium (*w*/*v*: 0.67% YNB, 0.5% esculin, 0.1% glucose, 0.02% ferric ammonium citrate, 2% agar; pH 5). A single colony was streaked onto the plate surface and incubated at 30 °C for 48–72 h. Strains showing beta-glucosidase activity produced a dark brown halo around it. Results were recorded as present or absent activity.

### 4.4. Fermentative Capacity of S. cerevisiae Strains in Synthetic Must

The *S. cerevisiae* strains were tested for their fermentative capacity on the synthetic must SM2 (Table S8), adjusted to pH 3.3 with HCl before use. Erlenmeyer flasks were sealed with a Müller valve containing sulfuric acid and filled with 160 mL of the synthetic must. The flasks were inoculated with the indigenous strains as axenic cultures to obtain an initial cell concentration of approximately 2 × 10^6^ cells/mL. The fermentations were carried out in duplicate at 25 °C. The progress of fermentation was monitored daily, recording weight loss, until the end of fermentation (when the weight remained constant for three consecutive days). The lag phase duration, the maximum fermentation rate and the maximum CO_2_ yield of each assayed strain were estimated by fitting the data to the Gompertz function. Substrates and products of the main metabolism of yeasts were detected at the end of the fermentations by High Performance Liquid Chromatography (HPLC), as reported below.

### 4.5. Lab-Scale Fermentation of S. cerevisiae Strains in Grape Must and Sensory Analysis

*S. cerevisiae* strains were inoculated as axenic cultures in Erlenmeyer flasks sealed with a Müller valve containing sulfuric acid and filled with 1 L of must obtained from Sangiovese grapes vinified without the skins (in white). The chemical composition of the grape must was as follows: total sugars, 227 g/L; malic acid, 1.08 g/L; NH_3_, 120 mg/L; αN, 30 mg/L; total SO_2_, 22 ppm; pH 3.60.

The flasks were inoculated to obtain an initial cell concentration of approximately 2 × 10^6^ CFU/mL. The fermentations were conducted, monitored, and statistically elaborated as reported above for the synthetic must test. At the end of alcoholic fermentation, the wines were analyzed by HPLC, as reported below, then transferred to glass bottles and stabilized at 4 °C for 7 days. After the stabilization, they were subjected to sensory analysis.

The sensory analysis was performed according to the methods reported by the Resolution OIV/CONCOURS 332A/2009 [29]. 15 trained panelists assessed the following descriptors: quality, frankness, intensity, persistence and general impression. All panelists evaluated the samples throughout one session in a randomized, blinded order using clear tasting glasses compliant with International Organization for Standardization (ISO) specifications, and they recorded their assessments for each descriptor on a scale ranging from “excellent” to “poor” assigning numerical values [53]. No sensitive data were collected during the sensory analysis, and no health risk was expected for the panelists.

### 4.6. Chemical Analysis

Glucose, fructose, ethanol, glycerol, 2,3-butanediol, acetic, citric, tartaric, lactic and succinic acid contents in must and wine were determined by HPLC, according to Guerrini et al. [54], utilizing a Rezex ROA-Organic Acid H+ (8%) column (8-μm particle, 300 × 7.8 mm; Phenomenex, Torrance, CA, USA) and a ProStar 210 chromatograph equipped with a DAD at 210 nm and a Refractive Index Detector, in series (Varian Inc., Palo Alto, CA, USA). Malic acid, NH3, and αN concentrations were determined enzymatically using an automated multi-parametric analyzer (Hyperlab, Steroglass, San Martino, Italy). For the determination of phenolic alcohols, wines were filtered (0.45 μm) and injected into the HPLC- UV/FLD Jasco series 4000 (Jasco, Japan Spectroscopic co, Hachioji city, Japan) equipped with a pump PU-4180, an autosampler AS-4050, a photodiode array detector MD-4010, and a column oven CO4060 and a reversed-phase column NovaPak C18 (4-μm particle, 300 × 3.9 mm; Waters, Milford, MA), thermostated at 25 °C. The mobile phase was (A) 2% (*v*/*v*) acetic acid in water and (B) acetonitrile; the gradient profile was 0–40 min, 1–20% B; 40–45 min, 20–50% B; and 45–55 min, 50–95% B, followed by washing with acetonitrile and re-equilibration of the column from 65 to 85 min; the flow rate was 0.9 mL/min from the beginning to 35 min and 1.0 mL/min from this point to the end. Phenolic alcohols were quantified at 280 nm using tyrosol and tryptophol as standards (Merck Life Science, Milano, Italy); hydroxytyrosol was expressed as tyrosol equivalents.

### 4.7. Proteomic Analysis

#### 4.7.1. Cell Cultures

Selected *S. cerevisiae* strains were precultured in YPD using rotary shaking (120 rpm) for 10 h at 30 °C. Then, 10^4^ cells/mL were transferred into synthetic must SM2 and incubated at 28 °C. Growth was evaluated daily by plating 100 µL aliquots of properly diluted cell suspensions on YPD-agar and counting CFUs after 3-day incubation at 30 °C. Three independent fermentations were run per strain, and cells were harvested at 40 h (early stage) and 76 h (mid-stage) for protein extraction. Culture supernatants for chemical analysis (glucose, fructose, ethanol and pyruvate) were also collected at 28, 52, 76, and 100 h. For protein extraction, cells were extensively washed with Milli-Q water and then with a lysis buffer (50 mM Mes, 10 mM EDTA, 10 mM MgCl_2_, pH 4.5). Each pellet was then resuspended in two volumes of lysis buffer supplemented with a protease inhibitor cocktail (Merck Life Science) and 0.1 M PMSF, then one volume of glass beads (425–600 μm, Merck Life Science) was added. Cell disruption was achieved by alternating 30 s of vigorous agitation with 30 s of rest in an ice bath for a total time of 8 min. After centrifugation (1000× *g*, 5 min, 4° C), the supernatants were collected, and their protein concentration was inferred by Bradford’s assay. Protein extracts were stored at −32 °C before 2D-PAGE.

#### 4.7.2. 2D-PAGE

Protein extracts obtained from the three independent fermentations were pooled for each strain and timepoint, so that each 2D gel represented the mean of three biological replicates. Protein extracts were first concentrated and desalted using Amicon Ultra centrifugal filters (Merck Millipore, Burlington, MA, USA). The protein samples were then solubilized in an isoelectric focusing (IEF) buffer consisting of 9 M urea, 65 mm CHAPS, 35 mm Tris, 65 mM dithioerythritol, and traces of bromophenol blue. Samples were diluted with an IPG rehydration solution (8 M urea, 30 mM CHAPS, 10 mM DTE, Resolyte pH 3.5–10, and traces of bromophenol blue) to a final volume of 700 µL. This solution was loaded onto 18 cm IPG strips (pH 3–10 nonlinear gradient; Bio-Rad, Hercules, CA, USA) via active rehydration for 16 h. First-dimension IEF was performed on a Multiphor II horizontal electrophoresis system (Amersham Biosciences, Uppsala, Sweden), while second-dimension SDS-PAGE was carried out in laboratory-made 9–16% gradient polyacrylamide gels. Both electrophoretic steps followed previously described protocols [55,56]. Following electrophoresis, gels were stained with colloidal Coomassie G-250 as described by Candiano et al. [57].

#### 4.7.3. Image Analysis

Digitized images of the Coomassie-stained gels were acquired with an ImageScanner III (Amersham Biosciences, Uppsala, Sweden) and analyzed using ImageMaster 2D Platinum software (v 7.0, Amersham Biosciences). Spot detection was followed by manual editing, and gels from the early and mid-timepoints of the same strain were matched pairwise. Spot abundance was expressed as relative volume (% volume), i.e., the volume of each spot normalized to the total volume of all valid spots in the gel, which compensates for differences in protein loading and staining intensity. Differentially abundant proteins (DAPs) were defined as those whose relative % volume ratio between the mid- and early timepoints was ≥ 2.0 (increased in abundance) or ≤ 0.5 (decreased in abundance). Proteins detected only at the mid-timepoint were classified as increased in abundance (P) and those detected only at the early timepoint as decreased in abundance (A). Because each gel represented a pool of three biological replicates, DAPs were identified by fold-change thresholds rather than by statistical testing. Protein spots were identified by gel-matching with in-house reference master gels [56] or by MALDI-ToF as described in Section 4.7.4.

#### 4.7.4. Matrix-Assisted Laser Desorption/Ionization—Time of Flight (MALDI-ToF)

2-D gels were rinsed with Milli-Q water before the manual excision of protein spots of interest. The excised spots were destained by washing twice with a 1:1 (*v*/*v*) solution of acetonitrile (ACN) and 5 mM ammonium bicarbonate for a minimum of 2 h, or until complete destaining was observed. Subsequently, the gel pieces were dehydrated by washing twice (10 min each) with pure ACN. Proteins were subjected to in-gel reduction and S-alkylation followed by digestion with mass spectrometry-grade trypsin (Promega, Madison, WI, USA).

The resulting peptide mixtures were deposited onto the MALDI target plate using the dried-droplet method, utilizing α-cyano-4-hydroxycinnamic acid (HCCA) as the matrix. Spectra were acquired using an ultrafleXtreme MALDI-ToF/ToF mass spectrometer (Bruker Daltonics, Leipzig, Germany) operating at a repetition rate of 1–2000 Hz, controlled by flexControl v.3.4 software. Internal mass calibration was performed using trypsin autolysis peaks.

Protein identification was carried out using flexAnalysis and the Mascot Peptide Mass Fingerprint (PMF) search engine (Matrix Science, London, UK) against an updated SwissProt *S. cerevisiae* database. Search parameters included cysteine carbamidomethylation as a fixed modification and methionine oxidation as a variable modification. Spots yielding low confidence scores in PMF were further subjected to MS/MS fragmentation analysis to confirm identification.

#### 4.7.5. Functional Enrichment Analysis

Lists of DAPs identified for each strain were submitted to the STRING database v12.0 [58,59] with *S. cerevisiae* as the reference organism. KEGG pathway enrichment was computed using the built-in enrichment tool against the whole *S. cerevisiae* proteome as statistical background, with *p* values corrected for multiple testing by the Benjamini–Hochberg procedure. Enrichment results were used descriptively, to summarize the functional categories represented among the DAPs.

#### 4.7.6. NMR Analysis of Supernatants

A 500 mM phosphate buffer (PB) was prepared from K_2_HPO_4_ and KH_2_PO_4_ (Merck KGaA, Darmstadt, Germany) in deionized water purified with a Milli-Q^®^ system (Millipore, Burlington, MA, USA), adjusted to pH 7.0 with 1 M NaOH. Each NMR sample consisted of 6 µL of culture supernatant, 30 µL of 10 mM trimethylsilylpropionic acid-d_4_ (TSP; Merck) to a final concentration of 0.5 mM, 60 µL of PB to a final concentration of 50 mM, and 504 µL of deuterium oxide (D_2_O, Merck), for a final volume of 600 µL, corresponding to a 100-fold dilution of the supernatant, which was accounted for in the reported concentrations.

Spectra were acquired on a Bruker Avance™ 600 spectrometer (Bruker BioSpin AG, Fällanden, Switzerland) operating at 14.1 T, using an optimized PURGE pulse sequence [60]. One-dimensional ^1^H spectra were recorded with a spectral width of 16 MHz, 8 scans, 6 k data points and a receiver gain of 512. The residual water signal was suppressed by pre-saturation (55 dB) applied during the 4 s relaxation delay. Chemical shifts were referenced to the TSP signal at 0.00 ppm. Spectra were processed with TopSpin 4.0.8 and analyzed with Chenomx 9.02 (Chenomx Inc., Edmonton, AB, Canada), which was used to identify and quantify the fermentation metabolites reported in Table S7.

### 4.8. Statistical Analysis

Kinetic parameters of the laboratory-scale fermentations were obtained by fitting cumulative CO_2_ production to a modified Gompertz function using GraphPad Prism 8 (GraphPad Software, San Diego, CA, USA). Chemical data, fermentation kinetic parameters and sensory scores were analyzed by one-way ANOVA followed by Tukey’s test, and Principal Component Analysis (PCA) was performed with Statistica 7.0 (StatSoft GmbH, Hamburg, Germany) for exploratory, unsupervised analysis of the samples subjected to chemical, microbiological and sensory characterization; no inferential testing was performed on the components.

To quantify the overall magnitude of the difference among strains for each metabolite, global effect size was evaluated using Eta-squared (η^2^), calculated via one-way Analysis of Variance (ANOVA). Values of η^2^ were interpreted according to standard benchmarks: small (≈0.01), medium (≈0.06), and large (≥0.14) [28]. Parameters with η^2^ ≥ 0.70, for which the strain factor accounted for at least 70% of the total variance, are indicated in bold in Tables S1 and S2. Given the limited number of replicates, η^2^ values are reported as descriptive indicators of the relative contribution of the strain factor rather than as unbiased estimates.

Viable counts from the proteomic experiment were log_10_-transformed before analysis and compared by two-way repeated-measures ANOVA, with time as the within-subject factor and strain as the between-subject factor, without assuming sphericity (Geisser–Greenhouse correction), followed by Tukey’s multiple comparisons test. These analyses were performed with GraphPad Prism 8.

Differences were considered significant at *p* < 0.05.

## 5. Conclusions

This study combined the oenological and the proteomic characterization of indigenous *S. cerevisiae* strains isolated from spontaneous fermentations in four Tuscan wineries. In both a synthetic and a Sangiovese grape must, the strains reached closely comparable ethanol yields while differing significantly in maximum fermentation rate and, in grape must, in the duration of the lag phase; the resulting wines nonetheless differed significantly in glycerol, acetic acid and tyrosol content, and in their sensory profiles. Proteomic comparison of four strains between an early and a mid-fermentation stage indicated that these differences did not reside in glycolysis, whose enzyme abundance varied without any corresponding variation in ethanol, but in the pathways branching from it: glycerol formation, acetaldehyde oxidation, sulfur amino acid metabolism and aromatic amino acid biosynthesis. Proteomic analysis therefore provided candidate molecular correlates of the traits on which the strains were distinguished, rather than predicting fermentation performance. Since indigenous populations have been reported to persist within individual cellars [4,9,13,14,15,16], this combination of approaches could be applied at the scale at which a tailored starter culture would be deployed.

## Figures and Tables

**Table 1:** Relative frequency (%) of *S. cerevisiae* strains in spontaneous wine fermentations carried out in four Tuscan wineries (A, B, C and D).

Winery	*S. cerevisiae* Strain	Frequency (%)
A	A_I	35
	A_III	32
B	B_I	15
	B_II	18
C	C_I	23
	C_XVIII	11
D	D_I	18
	D_II	20

**Table 2:** Kinetic parameters of fermentations carried out in synthetic must SM2 at 25 °C by indigenous *S. cerevisiae* strains inoculated at 2 × 10^6^ cells/mL. Parameters were estimated by fitting cumulative CO_2_ production to a modified Gompertz function: λ, duration of the lag phase; μ_max_, maximum fermentation rate; C, maximum cumulative CO_2_ production; R^2^, coefficient of determination of the fit [27]. Values are means ± standard deviation of two independent fermentations; values followed by different letters differ significantly (ANOVA and Tukey’s test, *p* < 0.05).

*S. cerevisiae* Strain	Lag Phase Mean ± SD (h)	µ_max_ Mean ± SD (dCO_2_/100 mL/h)	C Mean ± SD (CO_2_/100 mL)	R^2^
A_I	5 ± 2	(0.053 ± 0.001) ^a^	(9.3 ± 0.1) ^a^	0.992
A_III	7 ± 1	(0.054 ± 0.001) ^a^	(9.1 ± 0.0) ^a^	0.994
B_I	10 ± 1	(0.088 ± 0.001) ^b^	(7.3 ± 0.1) ^b^	0.985
B_II	12 ± 2	(0.101 ± 0.005) ^b^	(8.7 ± 0.5) ^a^	0.981
C_I	10 ± 1	(0.064 ± 0.002) ^a^	(10.0 ± 0.9) ^a^	0.984
C_XVIII	10 ± 1	(0.060 ± 0.001) ^a^	(8.6 ± 0.4) ^a^	0.987
D_I	10 ± 7	(0.062 ± 0.003) ^a^	(9.3 ± 0.4) ^a^	0.985
D_II	8 ± 2	(0.063 ± 0.008) ^a^	(10.1 ± 0.3) ^a^	0.987

**Table 3:** Chemical composition of experimental wines obtained from synthetic must fermentations by indigenous *S. cerevisiae* strains. Values are means ± standard deviation of two independent fermentations; values followed by different letters differ significantly (ANOVA and Tukey’s test, *p* < 0.05). Exact *p* values of the one-way ANOVA are reported for each parameter. Nd, not detectable; na, not applicable.

Parameter	A_I	A_III	B_I	B_II	C_I	C_XVIII	D_I	D_II	*p*
glucose (g/L)	nd	nd	(0.2 ± 0.1)	nd	nd	nd	(0.1 ± 0.1)	nd	na
fructose (g/L)	(2.3 ± 1.6) ^b^	(2.2 ± 0.9) ^b^	(6.9 ± 1.4) ^a^	(0.9 ± 0.4) ^b^	(0.7 ± 0.3) ^b^	(3.6 ± 0.3) ^ab^	(4.5 ± 0.1) ^a^	(0.6 ± 0.3) ^b^	0.0008
ethanol (% *v*/*v*)	(13.3 ± 0.2) ^ab^	(13.2 ± 0.1) ^ab^	(12.9 ± 0.1) ^a^	(13.4 ± 0.1) ^b^	(13.4 ± 0.1) ^b^	(13.1 ± 0.0) ^ab^	(13.1 ± 0.2) ^ab^	(13.4 ± 0.0) ^b^	0.0258
*Polyols*									
glycerol (g/L)	(4.1 ± 0.5) ^a^	(4.1 ± 0.1) ^a^	(4.4 ± 0.1) ^a^	(4.5 ± 0.1) ^a^	(4.5 ± 0.2) ^a^	(5.8 ± 0.2) ^b^	(4.2 ± 0.6) ^a^	(4.4 ± 0.1) ^a^	0.008
meso 2,3-butanediol (g/L)	(0.12 ± 0.01) ^b^	(0.10 ± 0.00) ^a^	(0.08 ± 0.01) ^a^	(0.14 ± 0.01) ^b^	(0.13 ± 0.01) ^b^	(0.19 ± 0.01) ^c^	(0.09 ± 0.01) ^a^	(0.14 ± 0.01) ^b^	<0.0001
racemic 2,3-butanediol (g/L)	(0.39 ± 0.01) ^d^	(0.37 ± 0.00) ^d^	(0.27 ± 0.01) ^a^	(0.49 ± 0.01) ^b^	(0.48 ± 0.01) ^b^	(0.58 ± 0.03) ^c^	(0.32 ± 0.02) ^a^	(0.46 ± 0.00) ^b^	<0.0001
1,2-propanediol (g/L)	(0.19 ± 0.04)	(0.20 ± 0.04)	(0.21 ± 0.06)	(0.27 ± 0.01)	(0.20 ± 0.06)	(0.23 ± 0.03)	(0.22 ± 0.03)	(0.21 ± 0.01)	0.6038
*Acids*									
succinic acid (g/L)	(0.32 ± 0.07)	(0.31 ± 0.06)	(0.48 ± 0.01)	(0.30 ± 0.07)	(0.38 ± 0.06)	(0.35 ± 0.04)	(0.37 ± 0.17)	(0.29 ± 0.07)	0.9991
acetic acid (g/L)	(0.60 ± 0.01) ^a^	(0.67 ± 0.02) ^a^	(0.61 ± 0.04) ^a^	(0.69 ± 0.01) ^a^	(0.75 ± 0.02) ^b^	(0.78 ± 0.02) ^b^	(0.59 ± 0.06) ^a^	(0.56 ± 0.04) ^a^	0.001
lactic acid (g/L)	(0.12 ± 0.02) ^ab^	(0.08 ± 0.01) ^a^	(0.09 ± 0.01) ^ab^	(0.08 ± 0.00) ^a^	(0.12 ± 0.01) ^ab^	(0.12 ± 0.02) ^ab^	(0.11 ± 0.01) ^ab^	(0.14 ± 0.01) ^b^	0.0116
*Phenolic alcohols*									
hydroxytyrosol (mg/L)	(0.76 ± 0.77)	(1.19 ± 0.08)	(0.97 ± 0.56)	(1.36 ± 0.01)	(1.72 ± 0.14)	(1.47 ± 0.31)	(1.45 ± 0.18)	(1.40 ± 0.18)	0.0849
tyrosol (mg/L)	(5.18 ± 0.55) ^ab^	(4.00 ± 0.25) ^a^	(5.23 ± 0.16) ^ab^	(4.41 ± 0.57) ^ab^	(6.48 ± 1.20) ^ab^	(9.71 ± 0.72) ^c^	(6.73 ± 0.80) ^a^	(6.09 ± 0.30) ^ab^	0.0005
tryptophol (mg/L)	(0.77 ± 0.14)	(0.78 ± 0.09)	(0.92 ± 0.06)	(0.62 ± 0.12)	(0.63 ± 0.14)	(1.07 ± 0.02)	(0.91 ± 0.33)	(1.11 ± 0.05)	0.0699

**Table 4:** Kinetic parameters of fermentations in Sangiovese grape must carried out by indigenous *S. cerevisiae* strains inoculated at 2 × 10^6^ cells/mL. Parameters were estimated by fitting cumulative CO_2_ production to a modified Gompertz function [27]: λ, duration of the lag phase; μ_max_, maximum fermentation rate; C, maximum cumulative CO_2_ production; R^2^, coefficient of determination of the fit. Values are means ± standard deviation of two independent fermentations; values followed by different letters differ significantly (ANOVA and Tukey’s test, *p* < 0.05).

*S. cerevisiae* Strain	Lag Phase (h)	µ_max_ (dCO_2_/100 mL/h)	C (CO_2_/100 mL)	R^2^
A_I	(12 ± 0) ^a^	(0.082 ± 0.002) ^a^	9.7 ± 0.1	0.985
A_III	(15 ± 0) ^ab^	(0.083 ± 0.001) ^a^	9.7 ± 0.1	0.993
B_I	(13 ± 2) ^a^	(0.095 ± 0.001) ^b^	9.8 ± 0.3	0.996
B_II	(11 ± 3) ^a^	(0.102 ± 0.005) ^bc^	9.6 ± 0.3	0.992
C_I	(25 ± 5) ^c^	(0.110 ± 0.008) ^bc^	9.2 ± 0.6	0.987
C_XVIII	(24 ± 1) ^bc^	(0.105 ± 0.006) ^bc^	9.3 ± 0.3	0.989
D_I	(17 ± 1) ^ab^	(0.115 ± 0.000) ^c^	9.7 ± 0.3	0.994
D_II	(12 ± 1) ^a^	(0.113 ± 0.004) ^c^	9.4 ± 0.1	0.994

**Table 5:** Chemical composition of experimental wines obtained from Sangiovese fermentations by indigenous *S. cerevisiae* strains. Values are means ± standard deviation of two independent fermentations; different letters indicate results differing significantly (ANOVA and Tukey’s test, *p* < 0.05). Exact *p* values of the one-way ANOVA are reported for each parameter. Nd, not detectable; na, not applicable.

Parameter	A_I	A_III	B_I	B_II	C_I	C_XVIII	D_I	D_II	*p*
glucose (g/L)	<0.1	<0.1	nd	nd	nd	nd	nd	nd	na
fructose (g/L)	(0.65 ± 0.21) ^bc^	(1.30 ± 0.42) ^c^	(0.45 ± 0.21) ^ab^	(0.20 ± 0.01) ^a^	(0.95 ± 0.21) ^ac^	(1.45 ± 0.11) ^c^	(0.55 ± 0.21) ^abc^	(0.55 ± 0.35) ^abc^	0.0100
ethanol (% *v*/*v*)	(13.35 ± 0.07)	(13.35 ± 0.07)	(13.25 ± 0.07)	(13.35 ± 0.07)	(13.25 ± 0.07)	(13.00 ± 0.00)	(13.30 ± 0.14)	(13.15 ± 0.21)	0.0921
*Polyols*									
glycerol (g/L)	(7.05 ± 0.21) ^ab^	(6.10 ± 0.14) ^a^	(6.65 ± 0.21) ^ab^	(6.60 ± 0.14) ^ab^	(6.45 ± 0.35) ^ab^	(8.05 ± 0.21) ^c^	(6.90 ± 0.14) ^ab^	(7.05 ± 0.21) ^b^	0.0005
meso 2,3-butanediol (g/L)	(0.19 ± 0.02) ^ab^	(0.12 ± 0.01) ^b^	(0.14 ± 0.01) ^ab^	(0.15 ± 0.01) ^ab^	(0.17 ± 0.01) ^ab^	(0.34 ± 0.03) ^c^	(0.14 ± 0.02) ^ab^	(0.15 ± 0.01) ^ab^	<0.0001
racemic 2,3-butanediol (g/L)	(0.49 ± 0.01) ^a^	(0.38 ± 0.00) ^a^	(0.37 ± 0.06) ^a^	(0.43 ± 0.01) ^a^	(0.52 ± 0.03) ^a^	(0.98 ± 0.07) ^b^	(0.44 ± 0.06) ^a^	(0.42 ± 0.04) ^a^	<0.0001
1,2-propanediol (g/L)	(0.23 ± 0.04) ^a^	(0.20 ± 0.01) ^a^	(0.19 ± 0.01) ^a^	(0.17 ± 0.01) ^a^	(0.19 ± 0.01) ^a^	(0.29 ± 0.02) ^b^	(0.18 ± 0.03) ^a^	(0.19 ± 0.02) ^a^	0.0089
*Acids*									
succinic acid (g/L)	(1.11 ± 0.06)	(0.79 ± 0.06)	(1.00 ± 0.11)	(1.00 ± 0.08)	(0.94 ± 0.06)	(0.79 ± 0.01)	(1.06 ± 0.04)	(1.17 ± 0.01)	0.9996
acetic acid (g/L)	(0.24 ± 0.01) ^ab^	(0.33 ± 0.01) ^ac^	(0.34 ± 0.09) ^a^	(0.16 ± 0.01) ^bd^	(0.26 ± 0.01) ^ab^	(0.45 ± 0.04) ^c^	(0.20 ± 0.02) ^ad^	(0.16 ± 0.03) ^bd^	0.0006
lactic acid (g/L)	(0.14 ± 0.01)	(0.17 ± 0.01)	(0.15 ± 0.01)	(0.17 ± 0.01)	(0.13 ± 0.01)	(0.17 ± 0.01)	(0.16 ± 0.03)	(0.17 ± 0.03)	0.2445
*Phenolic alcohols*									
hydroxytyrosol (mg/L)	(3.78 ± 0.02) ^ab^	(3.13 ± 0.03) ^b^	(3.86 ± 0.65) ^ab^	(4.70 ± 0.02) ^a^	(3.89 ± 0.16) ^ab^	(3.90 ± 0.38) ^ab^	(4.51 ± 0.19) ^a^	(4.73 ± 0.16) ^a^	0.0059
tyrosol (mg/L)	(26.40 ± 5.05) ^b^	(18.2 ± 3.60) ^a^	(16.9 ± 0.18) ^a^	(28.1 ± 0.67) ^b^	(33.5 ± 1.25) ^bc^	(22.9 ± 0.76) ^b^	(39.2 ± 0.80) ^cd^	(42.9 ± 0.35) ^d^	<0.0001
tryptophol (mg/L)	(1.93 ± 0.03) ^a^	(1.79 ± 0.02) ^a^	(2.03 ± 0.06) ^a^	(2.30 ± 0.14) ^a^	(2.72 ± 0.18) ^b^	(2.12 ± 0.05) ^a^	(3.54 ± 0.35) ^c^	(2.72 ± 0.07) ^b^	<0.0001

**Table 6:** Differentially abundant proteins in indigenous wine *S. cerevisiae* strains A_I, B_I, C_XVIII, and D_II during fermentation. Comparison of mid (76 h) versus early (40 h) fermentation stages was performed in fully synthetic wine must (SM2). Protein names, UniProt entry names, and UniProt IDs (https://www.uniprot.org/) are provided. Fold-changes were calculated as relative spot % volume ratios (mid vs. early time points). Fold-change values ≥ 2.0 (increased abundance) or ≤ 0.5 (decreased abundance) are highlighted in bold; nd: not detected; A: absent at mid fermentation; P: present only at mid fermentation.

Protein Name	UniProt Entry (_YEAST) *	UniProt ID	Strain			
			**A_I**	**B_I**	**C_XVIII**	**D_II**
Actin	ACT	P60010	1.2	0.6	1.1	**0.3**
Alcohol dehydrogenase 1 + Fructose-bisphosphate aldolase *	ADH1 + ALF	P00330 P14540	**P**	nd	**P**	nd
Adenosine kinase	ADK	P47143	**0.3**	0.8	0.8	0.9
Aldehyde dehydrogenase	ALDH2	P47771	**2.0**	**0.4**	**5.8**	0.6
Argininosuccinate lyase	ARYL	P04076	0.8	0.6	**0.4**	**0.5**
ATP synthase subunit beta, mitochondrial	ATPB	P00830	1.1	**0.5**	1.1	0.7
Endoplasmic reticulum chaperone BiP (78 kDa glucose-regulated protein homolog) (GRP-78)	BIP	P16474	0.6	**0.2**	**A**	0.8
Protein BMH1	BMH1	P29311	**0.2**	**0.5**	**0.5**	0.7
Protein BMH2	BMH2	P34730	**0.4**	1.4	**3.7**	0.7
Cystathionine beta-synthase	CBS	P32582	nd	nd	**2.6**	nd
Carboxypeptidase Y inhibitor	CPYI	P14306	1.4	1.8	0.6	**2.6**
Peptidyl-prolyl cis-trans isomerase D	CYPD	P35176	1.1	**5.9**	**2.3**	1.0
Cystathionine gamma-lyase	CYS3	P31373	nd	nd	**0.5**	nd
Homocysteine/cysteine synthase	CYSD	P06106	0.9	1.1	**2.0**	1.0
Deoxyhypusine hydroxylase	DOHH	P47120	**0.4**	**3.0**	**0.5**	0.6
Elongation factor 2	EF2	P32324	0.7	**0.5**	0.8	0.7
Enolase 1	ENO1	P00924	**2.4**	0.9	1.9	**3.0**
Farnesyl pyrophosphate synthase	ERG20	P08524	1.4	**2.5**	0.6	**P**
Sterol 24-C-methyltransferase ERG6	ERG6	P25087	0.8	**P**	1.5	1.4
FK506-binding protein 1	FKBP	P20081	1.4	**0.4**	**0.2**	**3.7**
Glyceraldehyde-3-phosphate dehydrogenase 1	G3P1	P00360	**0.4**	0.9	**P**	1.7
Glyceraldehyde-3-phosphate dehydrogenase 2	G3P2	P00358	1.0	**0.5**	1.0	1.8
Glyceraldehyde-3-phosphate dehydrogenase 2 + Glyceraldehyde-3-phosphate dehydrogenase 3 + Oxygen-dependent coproporphyrinogen-III oxidase *	G3P2 + G3P3 + HEM6	P00358 P00359 P11353	0.7	**2.8**	**2.4**	**0.3**
Glyceraldehyde-3-phosphate dehydrogenase 3	G3P3	P00359	**0.3**	**2.1**	1.4	1.2
Small ribosomal subunit protein RACK1	GBLP	P38011	0.6	0.7	0.6	**0.5**
Serine hydroxymethyltransferase, cytosolic	GLYC	P37291	nd	**P**	nd	nd
Glycerol-1-phosphate phosphohydrolase 1	GPP1	P41277	0.9	**0.4**	0.6	0.6
Glycerol-1-phosphate phosphohydrolase 2	GPP2	P40106	1.1	0.9	**2.1**	1.3
GTP-binding nuclear protein GSP1/CNR1	GSP1	P32835	0.8	**0.4**	0.7	1.6
ATP phosphoribosyltransferase	HIS1	P00498	**0.3**	1.6	1.5	**0.4**
Protein HRI1	HRI1	Q05905	nd	**0.3**	nd	nd
12 kDa heat shock protein	HSP12	P22943	0.7	**0.4**	**0.5**	**0.5**
Heat shock protein 60, mitochondrial	HSP60	P19882	**5.7**	**0.4**	**0.5**	**0.2**
Heat shock protein SSA2	HSP72	P10592	0.8	**0.2**	1.3	1.7
Import motor subunit, mitochondrial	HSP77	P0CS90	1.6	**0.5**	1.3	**0.3**
Heat shock protein homolog SSE1	HSP7F	P32589	**2.0**	**0.4**	**2.2**	**0.3**
Hexokinase-2	HXKB	P04807	**2.0**	0.6	0.6	**0.5**
Eukaryotic translation initiation factor 5A-1	IF5A1	P23301	1.0	0.7	0.7	**0.5**
Importin subunit alpha	IMA1	Q02821	**P**	**0.5**	**0.2**	nd
Inorganic pyrophosphatase	IPYR	P00817	1.2	**0.5**	1.5	0.7
Adenylate kinase	KAD2	P07170	1.5	**0.5**	0.8	1.2
Pyruvate kinase 1	KPYK1	P00549	nd	nd	**P**	**2.4**
3-isopropylmalate dehydrogenase	LEU3	P04173	1.1	0.8	1.2	**2.1**
Saccharopine dehydrogenase	LYS9	P38999	0.9	**0.4**	**0.4**	1.3
S-adenosylmethionine synthase 1	METK1	P10659	0.6	**0.3**	0.6	0.7
S-adenosylmethionine synthase 2	METK2	P19358	1.3	**0.4**	1.1	0.8
Protein MMF1, mitochondrial	MMF1	P40185	0.7	1.1	**0.2**	**3.3**
Pyruvate dehydrogenase E1 component subunit beta, mitochondrial	ODPB	P32473	**2.3**	**2.5**	1.8	**0.2**
Polyadenylate-binding protein, cytoplasmic and nuclear	PABP	P04147	**P**	**0.3**	1.0	**2.7**
Pyruvate decarboxylase isozyme 1	PDC1	P06169	**0.4**	0.7	0.6	0.9
ATP-dependent 6-phosphofructokinase subunit alpha	PFKA1	P16861	0.6	0.6	0.9	**0.5**
ATP-dependent 6-phosphofructokinase subunit beta	PFKA2	P16862	0.7	**0.4**	1.1	1.3
Phosphoglycerate mutase 1	PMG1	P00950	nd	**0.3**	**A**	nd
Phosphomannomutase	PMM	P07283	1.7	**0.3**	nd	nd
Pre-mRNA-processing protein 45	PRP45	P28004	nd	nd	nd	**A**
Proteasome subunit alpha type-2	PSA2	P23639	nd	**P**	nd	nd
Protoplast secreted protein 2	PST2	Q12335	1.1	**0.5**	0.9	0.8
Bifunctional purine biosynthetic protein ADE5,7 (includes: Phosphoribosylamine—glycine ligase; Phosphoribosylformylglycinamidine cyclo-ligase)	PUR2	P07244	nd	1.2	nd	**0.4**
Phosphoribosylaminoimidazole-succinocarboxamide synthase	PUR7	P27616	1.5	**2.1**	0.7	0.7
Large ribosomal subunit protein eL15B	RL15B	P54780	**2.2**	1.1	1.2	nd
Ubiquitin-ribosomal protein eL40A fusion protein (cleaved into: Ubiquitin; Large ribosomal subunit protein eL40A)	RL40A	P0CH08	**0.3**	**0.4**	**0.4**	**A**
Small ribosomal subunit protein uS11A	RS14A	P06367	nd	nd	nd	**3.4**
Small ribosomal subunit protein uS10	RS20	P38701	nd	nd	nd	**0.4**
Small ribosomal subunit protein eS21B	RS21B	Q3E754	0.8	**0.5**	1.7	1.3
Small ribosomal subunit protein eS4A	RS4A	P0CX35	**P**	0.9	**A**	nd
Small ribosomal subunit protein uS2A	RSSA1	P32905	nd	**P**	**P**	**0.5**
Small ribosomal subunit protein uS2B	RSSA2	P46654	**0.1**	0.6	1.0	1.1
SEC14 cytosolic factor	SEC14	P24280	**2.0**	**0.5**	1.4	0.9
Mitotic spindle-associated protein SHE1	SHE1	P38200	0.7	1.2	**0.5**	**P**
Heat shock protein STI1	STI1	P15705	nd	**P**	nd	nd
Glycine—tRNA ligase 1, mitochondrial	SYG	P38088	**2.2**	1.1	**0.3**	1.6
Lysine—tRNA ligase, cytoplasmic	SYKC	P15180	1.2	0.7	0.7	**3.6**
Transaldolase	TAL1	P15019	**0.4**	**7.3**	**0.4**	**2.3**
Tubulin beta chain	TBB	P02557	**A**	0.9	0.7	1.1
T-complex protein 1 subunit zeta	TCPZ	P39079	nd	**0.4**	1.2	nd
Translationally controlled tumor protein homolog	TCTP	P35691	**A**	0.6	0.9	0.6
Thiamine thiazole synthase	THI4	P32318	**0.5**	**0.5**	1.3	0.6
Tryptophan synthase	TRP	P00931	**2.2**	**2.9**	1.0	**5.8**
Ubiquitin carboxyl-terminal hydrolase YUH1	UBL1	P35127	nd	nd	nd	**P**
CTP synthase 1	URA7	P28274	nd	**0.4**	1.7	**4.0**
V-type proton ATPase catalytic subunit A	VATA	P17255	1.8	0.6	1.0	**0.4**
V-type proton ATPase subunit B	VATB	P16140	0.6	0.8	1.3	**0.3**
V-type proton ATPase subunit E	VATE	P22203	**2.2**	0.6	0.9	**2.2**
Transcriptional modulator WTM1	WTM1	Q12363	**P**	nd	nd	nd
NADP-dependent 3-hydroxy acid dehydrogenase	YM71	Q05016	nd	nd	**P**	nd

* Indicates co-migrated protein spots.

## Data Availability

All data supporting the findings of this study are contained within the article and its Supplementary Materials.

## Supplementary Materials

The following supporting information can be downloaded at https://www.mdpi.com/article/10.3390/ijms27219419/s1.

## Abbreviations

The following abbreviations are used in this manuscript:

2D-PAGE	Two-dimensional polyacrylamide gel electrophoresis
CFU	Colony-forming units
DAP	Differentially abundant protein
FC	Fold change
IEF	Isoelectric focusing
MALDI-ToF	Matrix-assisted laser desorption/ionization time-of-flight
OIV	International Organisation of Vine and Wine
PCA	Principal component analysis
SM2	Synthetic must 2
