# Supplementary material for: Differentiation of Indigenous Saccharomyces cerevisiae Wine Strains by Oenological and Proteomic Characterization
Source: Int J Mol Sci. 2026 Oct 22;27(21):9419. doi: 10.3390/ijms27219419 (PMC13762649; doi:10.3390/ijms27219419)
Supplement: Supplementary file 1 [file ijms-27-09419-s001.zip › ijms-4617855-supplementary.pdf]

Differentiation of indigenous *Saccharomyces cerevisiae* wine strains by oenological and proteomic characterization

Daniela Braconi [1,*], Simona Guerrini [2], Francesca Girolamo [1,#], Simone Costagli [1,§], Alessandro Paffetti [1], Alessandro Armini [1], Sara Draghi [1], Gabriella Jacomelli [1], Andrea Bernini [1], Daniela Grasso [1], Viola Galli [3], Donatella Ganucci [2], Lisa Granchi [2], Annalisa Santucci [1]

[1] Department of Biotechnology, Chemistry and Pharmacy (DBCF), University of Siena, via Aldo Moro 2, 53100 Siena, Italy;
[2] Department of Agriculture, Food, Environment and Forestry (DAGRI), University of Florence, Via San Bonaventura 13, 50145 Florence, Italy
[3] FoodMicroTeam s.r.l., ex Academic Spin-Off of the University of Florence, Via Santo Spirito 14, 50125 Florence, Italy

* Correspondence: Department of Biotechnology, Chemistry and Pharmacy (DBCF), University of Siena, 53100 Siena, Italy; e-mail daniela.braconi@unisi.it
# Current affiliation: Department of Molecular Medicine and Development, University of Siena, 53100 Siena, Italy
§ Current affiliation: Laboratory of Molecular Microbiology and Biotechnology, Department of Medical Biotechnologies, University of Siena, 53100 Siena, Italy

Supplementary Material

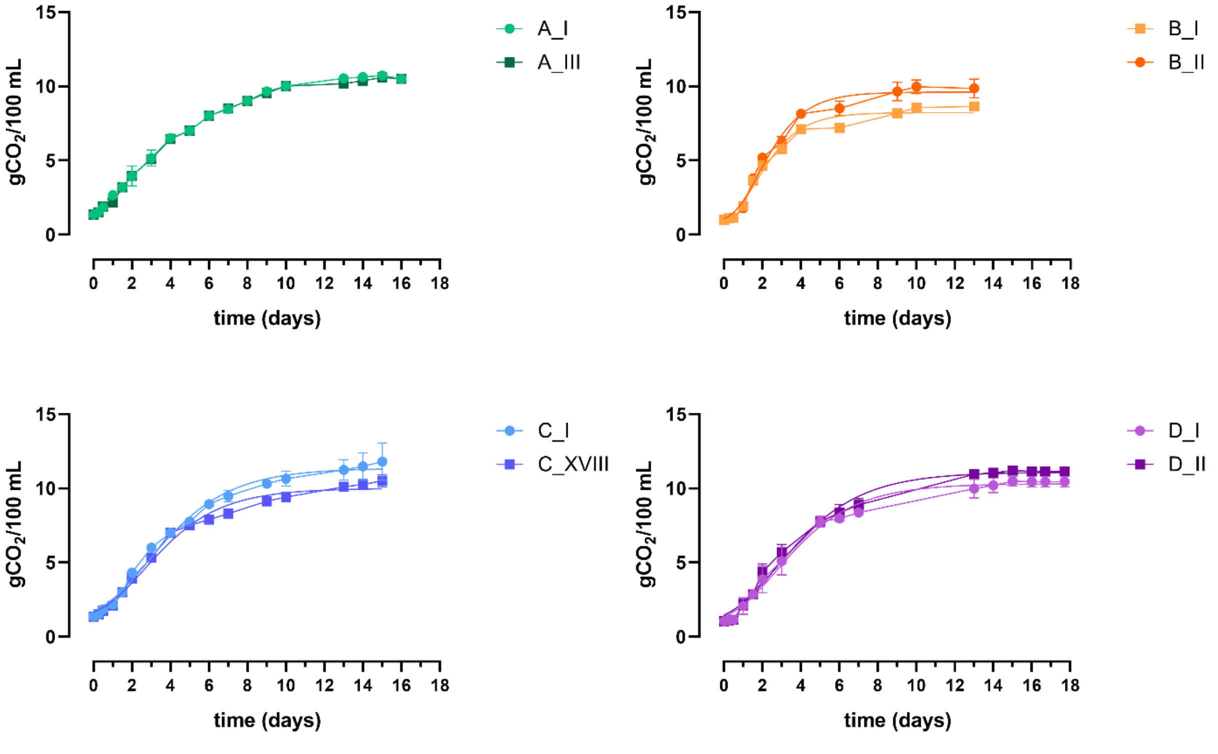

Figure S1. Cumulative CO_2 production during fermentation of synthetic must SM2 at 25 °C by the eight indigenous *S. cerevisiae* strains. Fermentation progress was followed by daily weight loss until constant weight over three consecutive days. Symbols represent means of two independent fermentations. Kinetic parameters derived by fitting these data to a modified Gompertz function [27] are given in Table 2 of the manuscript.

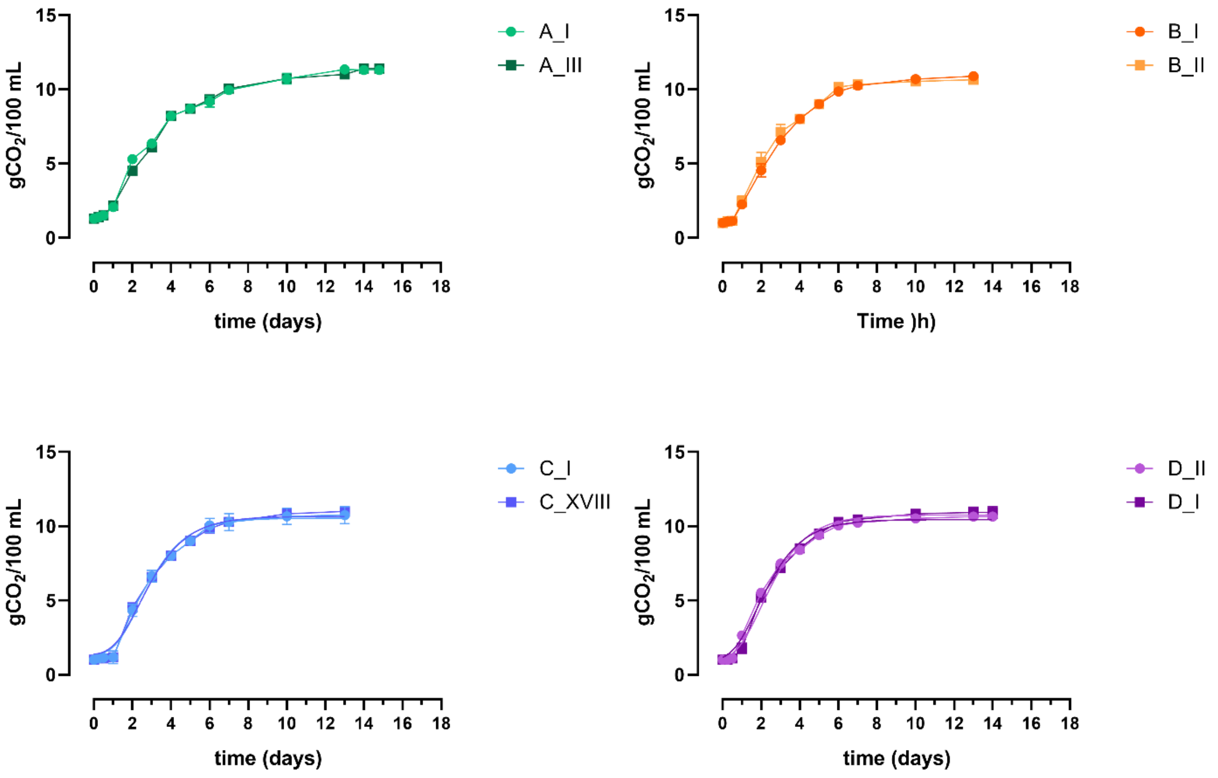

Figure S2. Cumulative CO_2 production during fermentation of Sangiovese grape must by the eight indigenous *S. cerevisiae* strains. Fermentation progress was followed by daily weight loss until constant weight over three consecutive days. Symbols represent means of two independent fermentations. Kinetic parameters derived by fitting these data to a modified Gompertz function [27] are given in Table 4 of the manuscript.

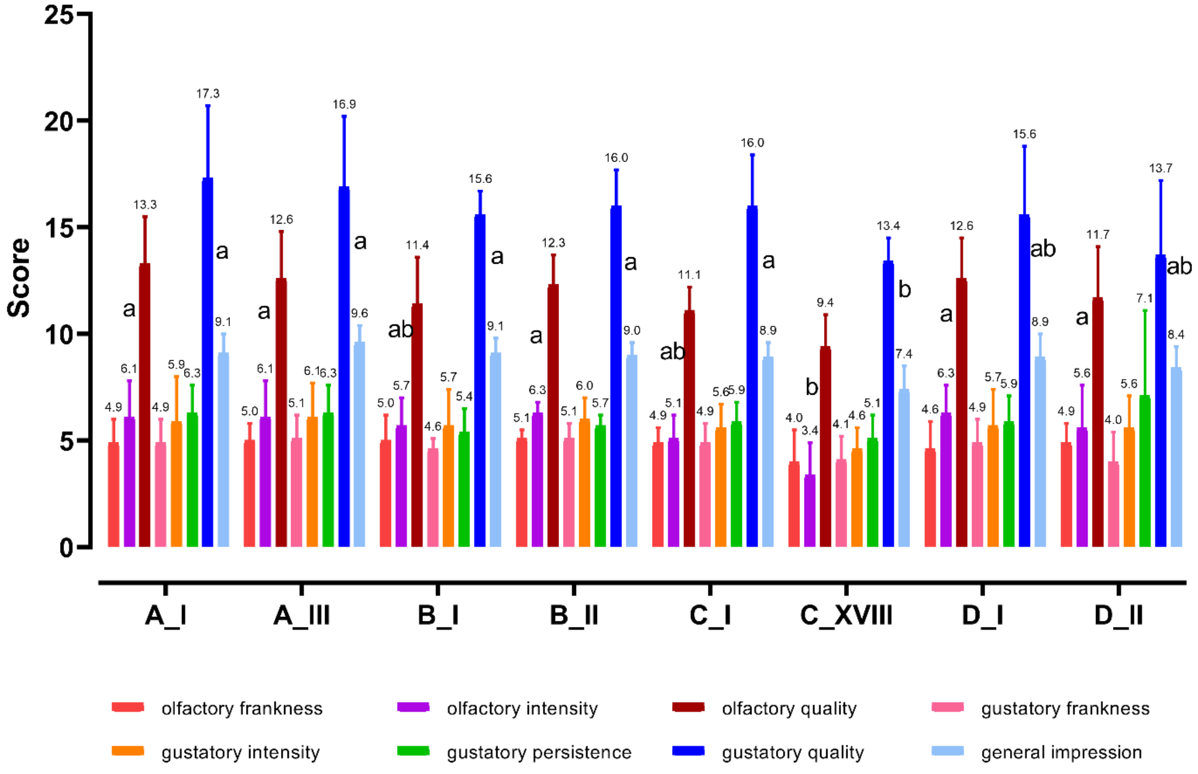

Figure S3. Sensory profiles of the eight experimental Sangiovese wines obtained by fermentation with the indigenous *S. cerevisiae* strains, assessed according to Resolution OIV/CONCOURS 332A/2009 [29]. Olfactory frankness, intensity and quality, gustatory frankness, intensity, persistence and quality, and general impression were scored by 15 trained panelists. All panelists evaluated the samples throughout one session in a randomized, blinded order using clear tasting glasses compliant with International Organization for Standardization (ISO) specifications, and they recorded their assessments for each descriptor on a scale ranging from “excellent” to “poor" assigning numerical values. For each descriptor, different letters indicate significant differences among wines (ANOVA followed by Tukey's test, $p < 0.05$).

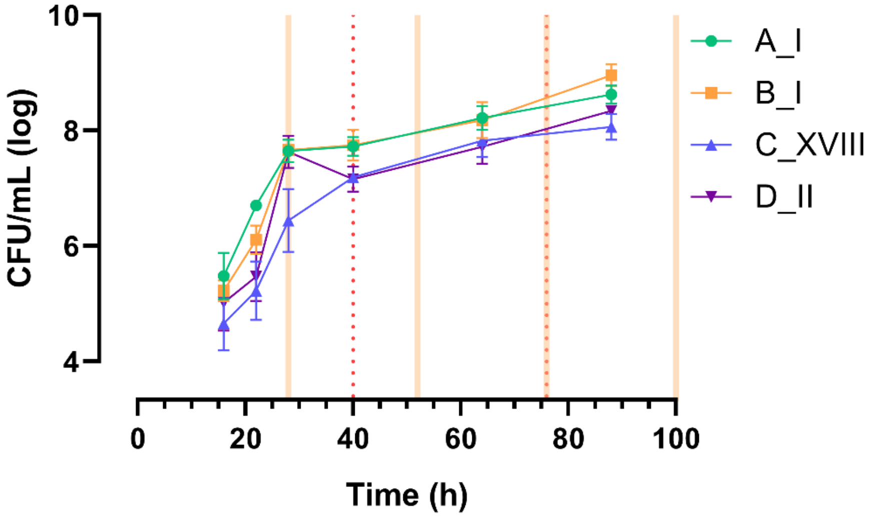

Figure S4. Growth of the indigenous *S. cerevisiae* strains A_I, B_I, C_XVIII and D_II during fermentation of synthetic must SM2 under the conditions used for proteomic analysis. Viable counts are expressed as log_{10} CFU/mL; symbols represent means of three independent fermentations and bars the standard deviation. Dashed red vertical lines mark the two sampling points used for proteomic analysis (40 h and 76 h), pink solid vertical lines mark the sampling points used for chemical analysis (as reported in Table S7). Data were analyzed by two-way repeated-measures ANOVA on log_{10}-transformed values, with time as the within-subject factor and strain as the between-subject factor and without assuming sphericity (Geisser–Greenhouse correction), followed by Tukey's multiple comparisons test; the only significant difference between strains at a single time point was between B_I and C_XVIII at 88 h ($p = 0.0220$).

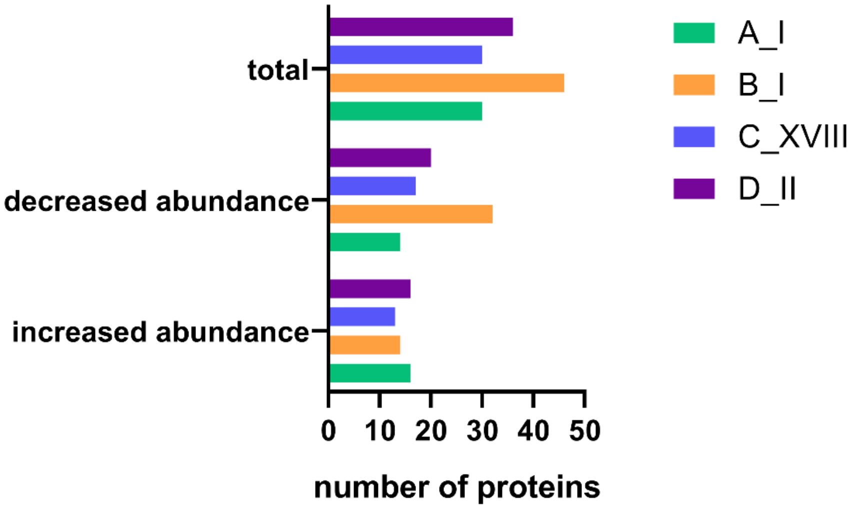

Figure S5. Differentially abundant proteins (DAPs) in indigenous wine *S. cerevisiae* strains A_I, B_I, C_XVIII, and D_II, comparing mid (76 h) to early (40 h) fermentation stages in fully synthetic wine must (SM2). Bars represent the number of proteins showing fold-changes ≥ 2.0 (increased abundance) or ≤ 0.5 (decreased abundance) calculated as relative spot % volume ratios (mid- vs. early time-points).

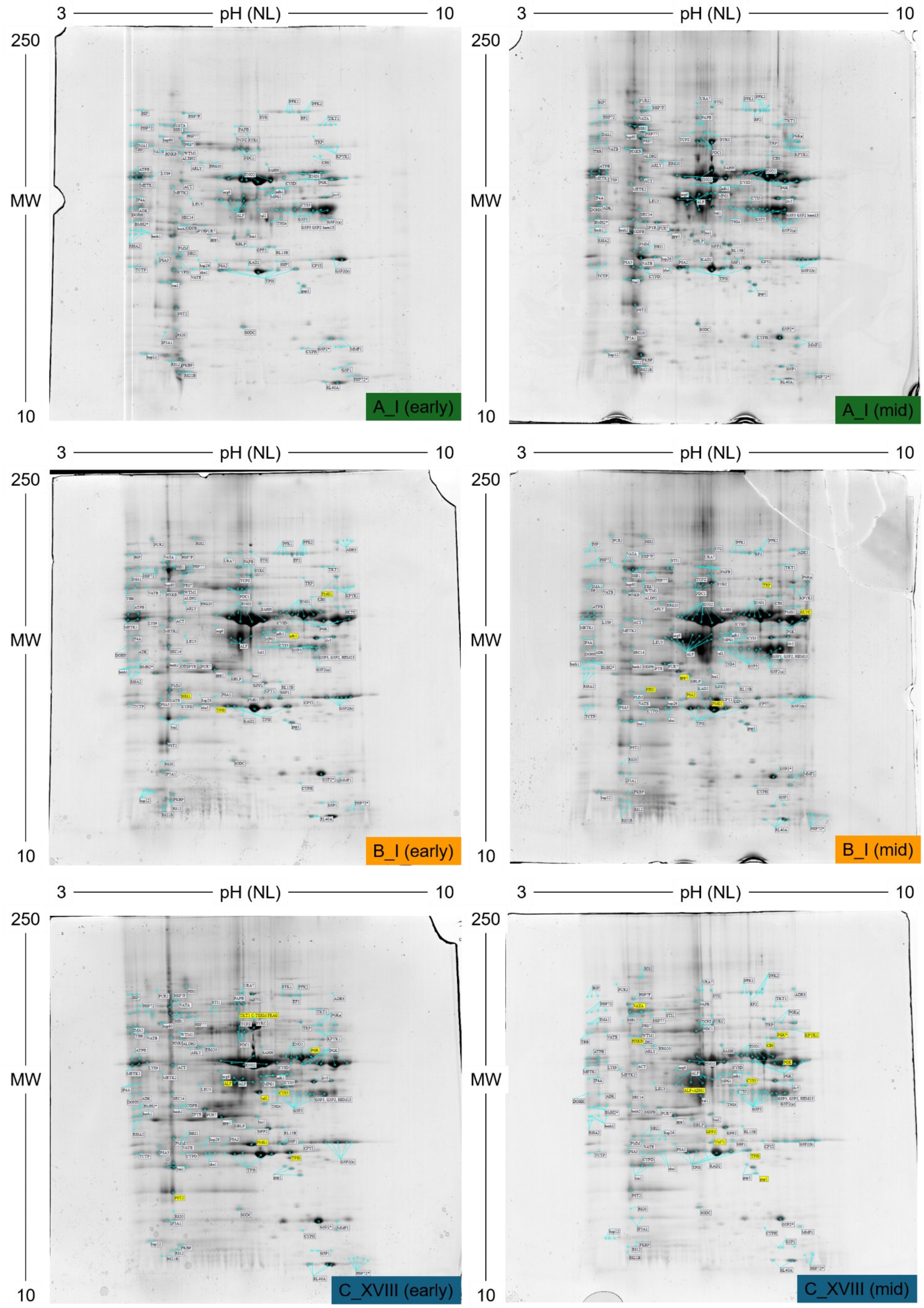
3
pH (NL)
10
250
MW
10
A_I (early)
A_I (mid)
B_I (early)
B_I (mid)
C_XVIII (early)
C_XVIII (mid)

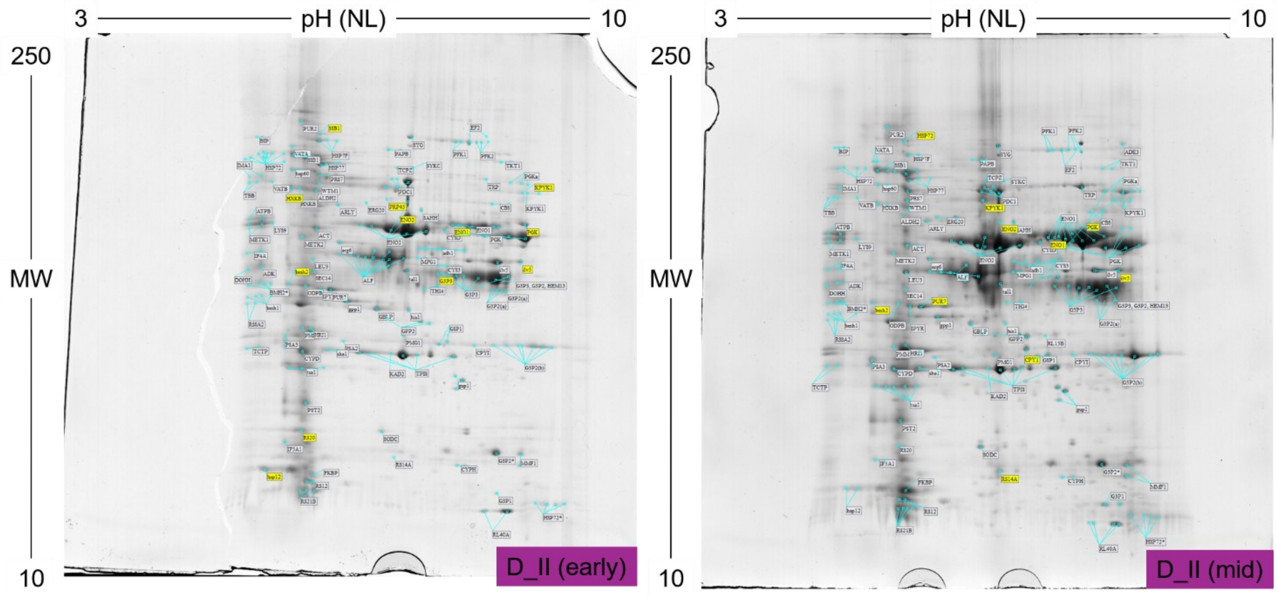

Figure S6. Two-dimensional gel maps of the indigenous *S. cerevisiae* strains A_I, B_I, C_XVIII and D_II at the early (40 h) and mid (76 h) fermentation stage in synthetic must SM2. Proteins were separated on 18 cm IPG strips (pH 3–10, non-linear) in the first dimension and on 9–16% gradient polyacrylamide gels in the second dimension, and visualized by colloidal Coomassie G-250 staining. Proteins are annotated with UniProt entries. Differentially abundant proteins (DAPs) are listed in Table 6 of the manuscript.

Table S1. Differences between replicates (Δ) and global effect size (GES) using eta-squared (η^2) calculated on the chemical composition of experimental wines obtained from synthetic must fermentations. Δ is the absolute difference between the two independent fermentations of each strain, expressed in the units of the corresponding parameter; GES (η^2) from one-way ANOVA is the proportion of the total variance in that parameter attributable to the strain factor, with $\eta^2 \geq 0.14$ corresponding to a large effect under conventional benchmarks [28]. Values of $\eta^2 \geq 0.70$, obtained here for 9 of the 12 parameters (in bold), correspond to at least 70% of the total variance being attributable to the strain factor, in contrast to the differences between replicates reported in the same row, and suggest a strong phenotypic differentiation among strains.

	**A_I**	**A_III**	**B_I**	**B_II**	**C_I**	**C_XVIII**	**D_I**	**D_II**	
**Parameter**	**Differences between replicates (Δ)**								**GES (η^2)**
**fructose (g/L)**	2.263	1.273	1.980	0.566	0.424	0.424	0.141	0.424	**0.921**
**ethanol (%v/v)**	0.283	0.141	0.141	0.141	0.141	0.000	0.283	0.000	**0.797**
**glycerol (g/L)**	0.707	0.141	0.141	0.141	0.283	0.283	0.849	0.141	**0.853**
**meso 2,3-butanediol (g/L)**	0.0141	0.0000	0.0141	0.0141	0.0141	0.0141	0.0141	0.0141	**0.961**
**racemic 2,3-butanediol (g/L)**	0.0141	0.0000	0.0141	0.0141	0.0141	0.0424	0.0283	0.0000	**0.988**
**1,2-propanediol (g/L)**	0.0566	0.0566	0.0849	0.0141	0.0849	0.0424	0.0424	0.0141	0.414
**succinic acid (g/L)**	0.0990	0.0849	0.0141	0.0990	0.0849	0.0566	0.2404	0.0990	0.505
**acetic acid (g/L)**	0.0141	0.0283	0.0566	0.0141	0.0283	0.0283	0.0849	0.0566	**0.915**
**lactic acid (g/L)**	0.0283	0.0141	0.0141	0.0000	0.0141	0.0283	0.0141	0.0141	**0.838**
**hydroxytyrosol (mg/L)**	1.088	0.1131	0.7920	0.0141	0.1980	0.4384	0.2546	0.2546	0.544
**tyrosol (mg/L)**	0.7778	0.3536	0.2263	0.8061	1.697	1.018	1.131	0.424	**0.929**
**Tryptophol (mg/L)**	0.1980	0.1273	0.0849	0.1697	0.1980	0.0283	0.4667	0.0707	**0.728**

Table S2. Differences between replicates (Δ) and global effect size (GES) using eta-squared (η^2) calculated on the chemical composition of experimental wines obtained from grape must fermentations. Δ is the absolute difference between the two independent fermentations of each strain, expressed in the units of the corresponding parameter; GES (η^2) from one-way ANOVA is the proportion of the total variance in that parameter attributable to the strain factor, with $\eta^2 \geq 0.14$ corresponding to a large effect under conventional benchmarks [28]. Values of $\eta^2 \geq 0.70$, obtained here for 11 of the 12 parameters (in bold), correspond to at least 70% of the total variance being attributable to the strain factor, in contrast to the differences between replicates reported in the same row, and suggest a strong phenotypic differentiation among strains.

	**A_I**	**A_III**	**B_I**	**B_II**	**C_I**	**C_XVIII**	**D_I**	**D_II**	
**Parameter**	**Differences between replicates (Δ)**								**GES (η^2)**
**fructose (g/L)**	0.2970	0.5940	0.2970	0.0141	0.2970	0.1556	0.2970	0.4950	**0.844**
**ethanol (%v/v)**	0.0990	0.0990	0.0990	0.0990	0.0990	0.0000	0.1980	0.2970	**0.704**
**glycerol (g/L)**	0.2970	0.1980	0.2970	0.1980	0.4950	0.2970	0.1980	0.2970	**0.929**
**meso 2,3-butanediol (g/L)**	0.0283	0.0141	0.0141	0.0141	0.0141	0.0424	0.0283	0.0141	**0.969**
**racemic 2,3-butanediol (g/L)**	0.0141	0.0000	0.0849	0.0141	0.0424	0.0990	0.0849	0.0566	**0.974**
**1,2-propanediol (g/L)**	0.0566	0.0141	0.0141	0.0141	0.0141	0.0283	0.0424	0.0283	**0.849**
**succinic acid (g/L)**	0.0849	0.0849	0.1556	0.1131	0.0849	0.0141	0.0566	0.0141	**0.896**
**acetic acid (g/L)**	0.0141	0.0141	0.1273	0.0141	0.0141	0.0566	0.0283	0.0424	**0.926**
**lactic acid (g/L)**	0.0141	0.0141	0.0141	0.0141	0.0141	0.0141	0.0424	0.0424	0.593
**hydroxytyrosol (mg/L)**	0.0283	0.0424	0.9192	0.0283	0.2263	0.5374	0.2687	0.2263	**0.865**
**tyrosol (mg/L)**	7.1418	5.0912	0.2546	0.9475	1.7678	1.0748	1.1314	0.4950	**0.968**
**tryptophol (mg/L)**	0.0424	0.0283	0.0849	0.1980	0.2546	0.0707	0.4950	0.0990	**0.961**

Table S3. KEGG pathway enrichment among the differentially abundant proteins of strain A_I, computed with STRING v12.0 against the whole *S. cerevisiae* proteome. Observed gene count, number of proteins from the input list annotated with the term; background gene count, number of proteins in the reference proteome carrying the term; strength, log_{10} of the observed/expected ratio; signal, weighted harmonic mean of the observed/expected ratio and −log(FDR); false discovery rate, p value corrected by the Benjamini–Hochberg procedure [58,59].

#term ID	term description	observed gene count	background gene count	strength	signal	false discovery rate	matching proteins in your network (IDs)	matching proteins in your network (labels)
sce00010	Glycolysis / Gluconeogenesis	9	54	1.55	2.56	6.75×10^{-10}	4932.YBR221C, 4932.YGL253W, 4932.YGR192C, 4932.YGR254W, 4932.YJL052W, 4932.YKL060C, 4932.YLR044C, 4932.YMR170C, 4932.YOL086C	PDB1, HXK2, TDH3, ENO1, TDH1, FBA1, PDC1, ALD2, ADH1
sce01110	Biosynthesis of secondary metabolites	12	344	0.87	0.99	1.51×10^{-6}	4932.YBR221C, 4932.YER055C, 4932.YGL026C, 4932.YGL253W, 4932.YGR192C, 4932.YGR254W, 4932.YJL052W, 4932.YKL060C, 4932.YLR044C, 4932.YLR354C, 4932.YMR170C, 4932.YOL086C	PDB1, HIS1, TRP5, HXK2, TDH3, ENO1, TDH1, FBA1, PDC1, TAL1, ALD2, ADH1
sce01100	Metabolic pathways	15	767	0.62	0.64	1.90×10^{-5}	4932.YBR221C, 4932.YER055C, 4932.YGL026C, 4932.YGL253W, 4932.YGR144W, 4932.YGR192C, 4932.YGR254W, 4932.YJL052W, 4932.YJR105W, 4932.YKL060C, 4932.YLR044C, 4932.YLR354C, 4932.YMR170C, 4932.YOL086C, 4932.YOR332W	PDB1, HIS1, TRP5, HXK2, THI4, TDH3, ENO1, TDH1, ADO1, FBA1, PDC1, TAL1, ALD2, ADH1, VMA4
sce01200	Carbon metabolism	7	113	1.12	1.15	2.76×10^{-5}	4932.YBR221C, 4932.YGL253W, 4932.YGR192C, 4932.YGR254W, 4932.YJL052W, 4932.YKL060C, 4932.YLR354C	PDB1, HXK2, TDH3, ENO1, TDH1, FBA1, TAL1
sce01230	Biosynthesis of amino acids	7	124	1.08	1.09	4.00×10^{-5}	4932.YER055C, 4932.YGL026C, 4932.YGR192C, 4932.YGR254W,	HIS1, TRP5, TDH3, ENO1, TDH1, FBA1, TAL1

							4932.YJL052W, 4932.YKL060C, 4932.YLR354C	
sce00340	Histidine metabolism	2	14	1.48	0.51	0.0459	4932.YER055C, 4932.YMR170C	HIS1, ALD2

Table S4. KEGG pathway enrichment among the differentially abundant proteins of strain B_I, computed with STRING v12.0 against the whole *S. cerevisiae* proteome. Observed gene count, number of proteins from the input list annotated with the term; background gene count, number of proteins in the reference proteome carrying the term; strength, $\log_{10}$ of the observed/expected ratio; signal, weighted harmonic mean of the observed/expected ratio and −log(FDR); false discovery rate, p value corrected by the Benjamini–Hochberg procedure [58,59].

#term ID	term description	observed gene count	background gene count	strength	signal	false discovery rate	matching proteins in your network (IDs)	matching proteins in your network (labels)
sce01110	Biosynthesis of secondary metabolites	19	344	0.9	1.35	7.78×10^{-11}	4932.YAR015W, 4932.YBR221C, 4932.YDR032C, 4932.YDR044W, 4932.YDR226W, 4932.YDR502C, 4932.YFL045C, 4932.YGL026C, 4932.YGR192C, 4932.YJL167W, 4932.YJR009C, 4932.YKL152C, 4932.YLR058C, 4932.YLR180W, 4932.YLR354C, 4932.YML008C, 4932.YMR170C, 4932.YMR205C, 4932.YNR050C	ADE1, PDB1, PST2, HEM13, ADK1, SAM2, SEC53, TRP5, TDH3, ERG20, TDH2, GPM1, SHM2, SAM1, TAL1, ERG6, ALD2, PFK2, LYS9
sce01100	Metabolic pathways	23	767	0.63	0.83	1.25×10^{-8}	4932.YAR015W, 4932.YBL039C, 4932.YBR221C, 4932.YDR032C, 4932.YDR044W, 4932.YDR226W, 4932.YDR502C, 4932.YFL045C, 4932.YGL026C, 4932.YGR144W, 4932.YGR192C, 4932.YIL053W, 4932.YJL167W, 4932.YJR009C, 4932.YJR121W, 4932.YKL152C, 4932.YLR058C, 4932.YLR180W, 4932.YLR354C, 4932.YML008C, 4932.YMR170C, 4932.YMR205C, 4932.YNR050C	ADE1, URA7, PDB1, PST2, HEM13, ADK1, SAM2, SEC53, TRP5, THI4, TDH3, GPP1, ERG20, TDH2, ATP2, GPM1, SHM2, SAM1, TAL1, ERG6, ALD2, PFK2, LYS9
sce01230	Biosynthesis of amino acids	10	124	1.06	1.31	6.57×10^{-7}	4932.YDR502C, 4932.YGL026C, 4932.YGR192C, 4932.YJR009C, 4932.YKL152C, 4932.YLR058C, 4932.YLR180W, 4932.YLR354C, 4932.YMR205C, 4932.YNR050C	SAM2, TRP5, TDH3, TDH2, GPM1, SHM2, SAM1, TAL1, PFK2, LYS9

sce00010	Glycolysis / Gluconeogenesis	6	54	1.2	1.11	8.39×10^{-5}	4932.YBR221C, 4932.YGR192C, 4932.YJR009C, 4932.YKL152C, 4932.YMR170C, 4932.YMR205C	PDB1, TDH3, TDH2, GPM1, ALD2, PFK2
sce01200	Carbon metabolism	7	113	0.95	0.83	0.00035	4932.YBR221C, 4932.YGR192C, 4932.YJR009C, 4932.YKL152C, 4932.YLR058C, 4932.YLR354C, 4932.YMR205C	PDB1, TDH3, TDH2, GPM1, SHM2, TAL1, PFK2
sce00680	Methane metabolism	3	25	1.24	0.57	0.017	4932.YKL152C, 4932.YLR058C, 4932.YMR205C	GPM1, SHM2, PFK2
sce03018	RNA degradation	4	63	0.96	0.51	0.0181	4932.YER165W, 4932.YJR045C, 4932.YLR259C, 4932.YMR205C	PAB1, SSC1, HSP60, PFK2
sce00260	Glycine, serine and threonine metabolism	3	30	1.16	0.53	0.0207	4932.YGL026C, 4932.YKL152C, 4932.YLR058C	TRP5, GPM1, SHM2

Table S5. KEGG pathway enrichment among the differentially abundant proteins of strain C_XVIII, computed with STRING v12.0 against the whole *S. cerevisiae* proteome. Observed gene count, number of proteins from the input list annotated with the term; background gene count, number of proteins in the reference proteome carrying the term; strength, log_{10} of the observed/expected ratio; signal, weighted harmonic mean of the observed/expected ratio and −log(FDR); false discovery rate, p value corrected by the Benjamini–Hochberg procedure [58,59].

#term ID	term description	observed gene count	background gene count	strength	signal	false discovery rate	matching proteins in your network (IDs)	matching proteins in your network (labels)
sce01230	Biosynthesis of amino acids	12	124	1.29	2.13	9.40×10^{-11}	4932.YAL012W, 4932.YAL038W, 4932.YGR155W, 4932.YGR192C, 4932.YHR018C, 4932.YJL052W, 4932.YJR009C, 4932.YKL060C, 4932.YKL152C, 4932.YLR303W, 4932.YLR354C, 4932.YNR050C	CYS3, CDC19, CYS4, TDH3, ARG4, TDH1, TDH2, FBA1, GPM1, MET17, TAL1, LYS9
sce01110	Biosynthesis of secondary metabolites	15	344	0.94	1.29	1.80×10^{-9}	4932.YAL012W, 4932.YAL038W, 4932.YDR044W, 4932.YGR155W, 4932.YGR192C, 4932.YHR018C, 4932.YJL052W, 4932.YJR009C, 4932.YKL060C, 4932.YKL152C, 4932.YLR303W, 4932.YLR354C, 4932.YMR170C, 4932.YNR050C, 4932.YOL086C	CYS3, CDC19, HEM13, CYS4, TDH3, ARG4, TDH1, TDH2, FBA1, GPM1, MET17, TAL1, ALD2, LYS9, ADH1
sce00010	Glycolysis / Gluconeogenesis	8	54	1.47	2.16	1.59×10^{-8}	4932.YAL038W, 4932.YGR192C, 4932.YJL052W, 4932.YJR009C, 4932.YKL060C, 4932.YKL152C, 4932.YMR170C, 4932.YOL086C	CDC19, TDH3, TDH1, TDH2, FBA1, GPM1, ALD2, ADH1
sce01100	Metabolic pathways	17	767	0.65	0.73	7.61×10^{-7}	4932.YAL012W, 4932.YAL038W, 4932.YDR044W, 4932.YER062C, 4932.YGR155W, 4932.YGR192C, 4932.YHR018C, 4932.YJL052W, 4932.YJR009C, 4932.YKL060C, 4932.YKL152C, 4932.YLR303W, 4932.YLR354C, 4932.YMR170C, 4932.YMR226C, 4932.YNR050C, 4932.YOL086C	CYS3, CDC19, HEM13, GPP2, CYS4, TDH3, ARG4, TDH1, TDH2, FBA1, GPM1, MET17, TAL1, ALD2, YMR226C, LYS9, ADH1

sce01200	Carbon metabolism	8	113	1.15	1.35	2.08×10^{-6}	4932.YAL038W, 4932.YGR192C, 4932.YJL052W, 4932.YJR009C, 4932.YKL060C, 4932.YKL152C, 4932.YLR303W, 4932.YLR354C	CDC19, TDH3, TDH1, TDH2, FBA1, GPM1, MET17, TAL1
sce00260	Glycine, serine and threonine metabolism	4	30	1.43	1.12	0.0004	4932.YAL012W, 4932.YGR155W, 4932.YKL152C, 4932.YMR226C	CYS3, CYS4, GPM1, YMR226C
sce00270	Cysteine and methionine metabolism	3	43	1.14	0.54	0.0238	4932.YAL012W, 4932.YGR155W, 4932.YLR303W	CYS3, CYS4, MET17
sce00310	Lysine degradation	2	16	1.4	0.48	0.0494	4932.YMR170C, 4932.YNR050C	ALD2, LYS9

Table S6. KEGG pathway enrichment among the differentially abundant proteins of strain D_II, computed with STRING v12.0 against the whole *S. cerevisiae* proteome. Observed gene count, number of proteins from the input list annotated with the term; background gene count, number of proteins in the reference proteome carrying the term; strength, $\log_{10}$ of the observed/expected ratio; signal, weighted harmonic mean of the observed/expected ratio and −log(FDR); false discovery rate, p value corrected by the Benjamini–Hochberg procedure [58,59].

#term ID	term description	observed gene count	background gene count	strength	signal	false discovery rate	matching proteins in your network (IDs)	matching proteins in your network (labels)
sce01110	Biosynthesis of secondary metabolites	15	344	0.88	1.13	4.15×10^{-8}	4932.YAL038W, 4932.YBR221C, 4932.YCL018W, 4932.YDR044W, 4932.YER055C, 4932.YGL026C, 4932.YGL234W, 4932.YGL253W, 4932.YGR192C, 4932.YGR240C, 4932.YGR254W, 4932.YHR018C, 4932.YJL167W, 4932.YJR009C, 4932.YLR354C	CDC19, PDB1, LEU2, HEM13, HIS1, TRP5, ADE5, 7, HXK2, TDH3, PFK1, ENO1, ARG4, ERG20, TDH2, TAL1
sce01230	Biosynthesis of amino acids	10	124	1.15	1.5	1.33×10^{-7}	4932.YAL038W, 4932.YCL018W, 4932.YER055C, 4932.YGL026C, 4932.YGR192C, 4932.YGR240C, 4932.YGR254W, 4932.YHR018C, 4932.YJR009C, 4932.YLR354C	CDC19, LEU2, HIS1, TRP5, TDH3, PFK1, ENO1, ARG4, TDH2, TAL1
sce01100	Metabolic pathways	19	767	0.63	0.75	2.98×10^{-7}	4932.YAL038W, 4932.YBL039C, 4932.YBR127C, 4932.YBR221C, 4932.YCL018W, 4932.YDL185W, 4932.YDR044W, 4932.YER055C, 4932.YGL026C, 4932.YGL234W, 4932.YGL253W, 4932.YGR192C, 4932.YGR240C, 4932.YGR254W, 4932.YHR018C, 4932.YJL167W, 4932.YJR009C, 4932.YLR354C, 4932.YOR332W	CDC19, URA7, VMA2, PDB1, LEU2, VMA1, HEM13, HIS1, TRP5, ADE5, 7, HXK2, TDH3, PFK1, ENO1, ARG4, ERG20, TDH2, TAL1, VMA4
sce00010	Glycolysis / Gluconeogenesis	7	54	1.35	1.63	1.11×10^{-6}	4932.YAL038W, 4932.YBR221C, 4932.YGL253W, 4932.YGR192C, 4932.YGR240C, 4932.YGR254W, 4932.YJR009C	CDC19, PDB1, HXK2, TDH3, PFK1, ENO1, TDH2

sce01200	Carbon metabolism	8	113	1.09	1.21	6.76×10^{-6}	4932.YAL038W, 4932.YBR221C, 4932.YGL253W, 4932.YGR192C, 4932.YGR240C, 4932.YGR254W, 4932.YJR009C, 4932.YLR354C	CDC19, PDB1, HXK2, TDH3, PFK1, ENO1, TDH2, TAL1
sce03018	RNA degradation	5	63	1.14	0.9	0.00071	4932.YER165W, 4932.YGR240C, 4932.YGR254W, 4932.YJR045C, 4932.YLR259C	PAB1, PFK1, ENO1, SSC1, HSP60
sce04145	Phagosome	3	35	1.17	0.55	0.0205	4932.YBR127C, 4932.YDL185W, 4932.YOR332W	VMA2, VMA1, VMA4

Table S7. Concentrations of hexoses, ethanol and pyruvate during fermentation of synthetic must SM2 by the indigenous *S. cerevisiae* strains A_I, B_I, C_XVIII and D_II under the conditions used for proteomic analysis. Samples were taken at 28, 52, 76, and 100 h and analyzed by NMR. Values are means of three independent fermentations; the coefficient of variation of the analytical method was ≤ 5% for all analytes.

		mean			
	**time**	**A_I**	**B_I**	**C_XVIII**	**D_II**
glucose+fructose (g/L)	28 h	197.7	196.9	198.6	200.9
	52 h	185.6	178.4	163.2	172.1
	76 h	143.9	145.1	139.3	138.6
	100 h	112.5	121.2	108.8	114.2
ethanol (g/L)	28 h	0.34	0.29	1.26	0.36
	52 h	3.41	2.70	5.03	6.71
	76 h	6.70	13.96	15.02	8.01
	100 h	11.59	14.14	11.18	15.15
pyruvate (g/L)	28 h	0.02	0.02	0.03	0.02
	52 h	0.11	0.09	0.1	0.09
	76 h	0.12	0.11	0.12	0.13
	100 h	0.13	0.11	0.14	0.15

Table S8. Composition of the synthetic must SM2, modified from Henschke and Jiranek [26] and adopted by the OIV for the characterization of *Saccharomyces* wine yeasts [25]. The medium was adjusted to pH 3.3 with HCl and sterilized by filtration.

	Component	Amount per litre
Carbon sources	Glucose	115 g
	Fructose	115 g
Organic Acids	Potassium tartrate	5 g
	Citric acid	0.2 g
	L-malic acid	3 g
Salts	K_2HPO_4	1.14 g
	$MgSO_4·7H_2O$	1.23 g
	$CaCl_2·2H_2O$	0.44 g
	$MnCl_2·4H_2O$	198.2 μg
	$ZnCl_2$	135.5 μg
	$CuCl_2$	13.6 μg
	$FeCl_2$	32.0 μg
	H_3BO_3	5.7 μg
	$Co(NO_3)_2·6H_2O$	29.1 μg
	$NaMoO_4·2H_2O$	24.2 μg
	KIO_3	10.8 μg
Vitamins	Myo-inositol	20 mg
	Pyridoxine hydrochloride	0.40 mg
	Nicotinic acid	0.40 mg
	Calcium pantothenate	0.20 mg
	Thiamine hydrochloride	0.10 mg
	p-Aminobenzoic acid	0.04 mg
	Riboflavin	0.04 mg
	Biotin	0.03 mg
	Folic acid	0.04 mg
Amino Acids	Aspartic acid	89 mg
	Glutamic acid	126 mg
	Alanine	26 mg
	Arginine	188 mg
	Asparagine	39 mg
	Phenylalanine	39 mg
	Glycine	14 mg
	Glutamine	51 mg
	Isoleucine	51 mg
	Histidine	39 mg
	Leucine	76 mg
	Lysine	63 mg
	Methionine	39 mg
	Proline	126 mg
	Serine	101 mg
	Tyrosine	6.00 mg
	Threonine	89 mg
	Tryptophan	26 mg
	Valine	51 mg
Nitrogen source	$(NH_4)2HPO_4$	100 mg
Lipid	Ergosterol	10 mg
Detergent	Tween 80®	0.5 mL
